# Over-Actuated Underwater Robots: Configuration Matrix Design and Perspectives †

**DOI:** 10.3390/s21227729

**Published:** 2021-11-20

**Authors:** Tho Dang, Lionel Lapierre, Rene Zapata, Benoit Ropars, Pascal Lepinay

**Affiliations:** 1 Laboratory of Informatics, Robotics and MicroElectronics (LIRMM) (UMR 5506 CNRS—UM), Université Montpellier, 161 rue Ada, CEDEX 5, 34392 Montpellier, France; zapata@lirmm.fr (R.Z.); lepinay@lirmm.fr (P.L.); 2 Reeds Company, 199 rue Hélène Boucher, 34170 Castelnau-Le-Lez, France; ropars@lirmm.fr

**Keywords:** over-actuated underwater robots, multi-objective optimization, underwater robots, performance indices

## Abstract

In general, for the configuration designs of underwater robots, the positions and directions of actuators (i.e., thrusters) are given and installed in conventional ways (known points, vertically, horizontally). This yields limitations for the capability of robots and does not optimize the robot’s resources such as energy, reactivity, and versatility, especially when the robots operate in confined environments. In order to optimize the configuration designs in the underwater robot field focusing on over-actuated systems, in the paper, performance indices (manipulability, energetic, reactive, and robustness indices) are introduced. The multi-objective optimization problem was formulated and analyzed. To deal with different objectives with different units, the goal-attainment method, which can avoid the difficulty of choosing a weighting vector to obtain a good balance among these objectives, was selected to solve the problem. A solution design procedure is proposed and discussed. The efficiency of the proposed method was proven by simulations and experimental results.

## 1. Introduction

**Figure 1:**
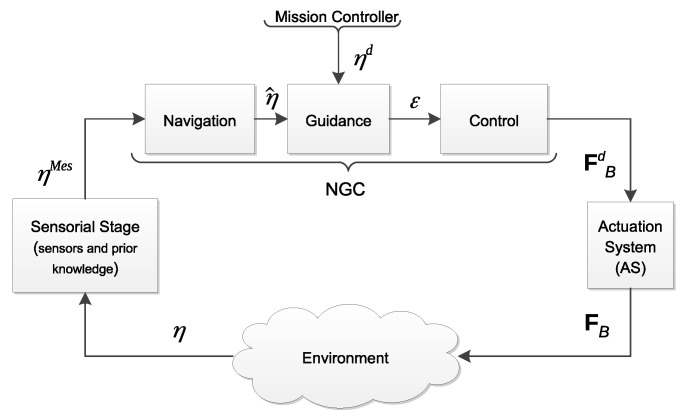
NGC structure augmented with the actuation system and sensorial stage.

The *Actuation System* (AS) is an important part of marine robots. The AS groups the different actuators carried by the system. Following the generic Navigation–Guidance–Control (NGC) structure, the AS is in charge of realizing the desired force ($F_{B}^{d}$) provided by the control system (see Figure 1).

**Figure 2:**
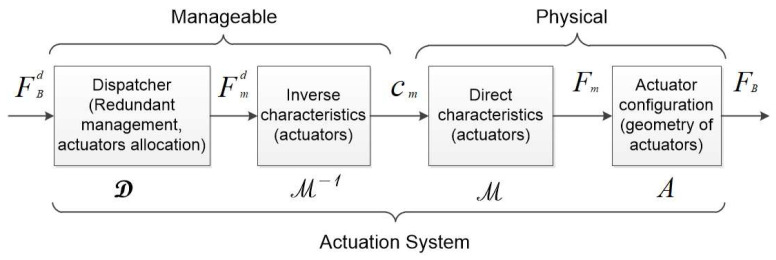
Actuation system scheme.

Following Figure 1, the sensorial stage using sensors measurement and prior knowledge of the environment provides to the navigation system the necessary information to compute an estimation of the system state ($\hat{\eta }$). Then, the guidance system uses this estimation and the reference system state ($\eta^{d}$) provided by the mission controller to compute the error function (ε). The control system is then in charge of computing the desired force ($F_{B}^{d}$) in order to reduce the error function to zero. Note that classically, this desired force is expressed in the body-frame. Afterwards, the *actuation system* produces in the environment a resulting force ($F_{B}$), which should be as close as possible to $F_{B}^{d}$. Note that, in this paper, the desired force ($F_{B}^{d}$) and resulting force ($F_{B}$) are (6×1) vectors and include force and torque elements. Inside the AS block, referring to Figure 2, the desired force ($F_{B}^{d}$) is the output of the controller. Then, the dispatcher (D) considers the actuator allocation method (and eventually, redundancy management) to compute the desired actuator force ($F_{m}^{d}$) that each actuator has to produce. The inverse actuator characteristics are then considered in order to compute the actuator inputs ($c_{m}$). Once applied, $c_{m}$ can produce actuator forces ($F_{m}$). The resulting force $F_{B}$ is produced with respect to the actuator configuration (A). The properties of the AS are indeed dependent on the actuator configuration (position and attitude of the actuators with respect to the body-frame), actuator dynamics (response characteristics), and dispatcher (control allocation, redundancy management) (see Figure 2) and afford the system different properties. Let us consider in the following that *n* is the number of Degrees of Freedom (DoFs) of the system and *m* is the number of actuators. If the system carries less actuators than DoFs, it is said to be *underactuated* (in that case, A will be an (n×m) matrix where n>m). Long-range Autonomous Underwater Vehicles (AUVs) and, for the terrestrial case, unicycle wheeled vehicles belong to this category [1]. In that case, specific nonlinear guidance strategies have to be used [2]. If the system carries more actuators than DoFs, it is said to be *redundant* (n<m). Then, there are different solutions ($c_{m}$) to produce an identical resulting force ($F_{B}$). Indeed, D is one of the multiple possible inverses of A, classically $D=A^{+}$, where $A^{+}$ is the Moore–Penrose pseudo-inverse. The properties of the AS play a pivotal role in the system performances, in terms of achievable dynamics, maneuverability, robustness, and dependability. The properties of an *overactuated* system have been studied in aerospace control, where critical safety is required [3], and for marine vehicles [4], where the harsh oceanic condition may easily produce actuator failure. Redundancy was also used in [5] in order to compensate different and unknown actuator responses. The domain of robotic manipulators has also extensively studied this question of redundancy, especially with recent works on humanoid robotics, where the task function approach [6] has been used to concurrently achieve equilibriums [7], walking pattern following [8], and multicontact management [9].

For a global evaluation of an *actuation system*, we should of course consider many factors, including redundancy management, the control allocation method, the actuator characteristics (inverse and direct), and the actuator configuration. This paper focuses on the study of the actuator configuration; for other problems, the readers can refer to [5] and the references therein.

Different performance criteria related to the actuator configuration design have been proposed. For mobile manipulation, the *manipulability index* [10] measures the manipulation capability of the end-effector. Intuitively, this index regards the set of all end-effector velocities, which is realizable by joint velocities. This set is called the hyper-manipulability ellipsoid. This index is quantified by computing the hyper-manipulability ellipsoid’s properties. Based on these properties, there are different ways to quantify the manipulability index, including the volume of the hyper-manipulability ellipsoid, the ratio of the minimum and maximum radii of the hyperellipsoid, and the minimum radius of the hyperellipsoid. The selection depends on the purpose of evaluation. When the uniformity of manipulating ability is important, the ratio of two radii of the hyperellipsoid is chosen (the optimal value will be close to one). Otherwise, the minimum radius of the hyperellipsoid is suited for the case where the minimum manipulating ability might be critical [11]. Another criterion, *attainability* [12,13,14], was studied using workspace volume estimation.

In the underwater robotics field, the *manipulability index*, *energetic index*, and *force index* were introduced in [15], and the manipulability index was applied in [16]. Specifically, the *manipulability index* is used to measure the system’s ability to exert a desired force with a specific actuator configuration. Therefore, the closer to one this index is, the better the robot’s isotropy is, i.e., the robot can exert the same forces/torques in any direction. The *energetic index* is a measurement of the variation of system energy when the direction of the desired force changes. This is evaluated by measuring the energy consumption when the direction of a normalized desired force changes over a 3D sphere. The basic idea of the energetic index is to keep the system’s energy consumption constant and as low as possible when the direction of action changes. The *force index* is used to measure the ratio between the actual maximum value and the minimum value of realizing forces. However, these studies only considered a given and fixed actuator configuration. Regarding the design of the actuator configuration of an overactuated underwater robot, a general problem is how to achieve an optimal configuration considering different performance indices. This is a challenging issue that raises two specific questions:

1. How do we define general and typical indices to evaluate an actuator configuration of an overactuated underwater robot?
2. How do we solve the complex optimal problem, which is normally nonconvex and has some conflicting objectives?

This paper focuses on the design of the actuator configuration for an overactuated underwater robot with the contributions outlined below:

1. We propose performance indices to evaluate the actuator configuration of underwater robots;
2. We optimize an actuator configuration design of an overactuated underwater robot with respect to different performance indices simultaneously.

**Figure 3:**
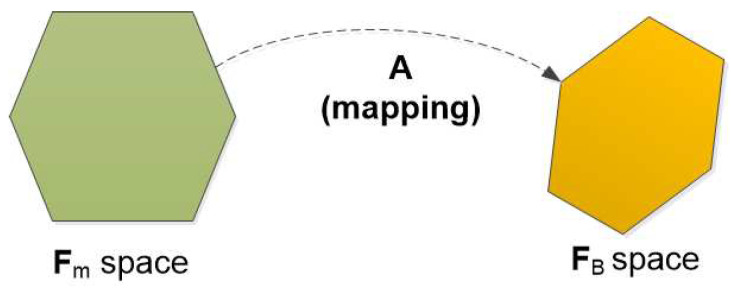
Actuator configuration mapping.

This paper focuses on the design of an actuator configuration of an overactuated underwater robot, which optimizes different performance indices. Mathematically, an actuator configuration is a mapping from an actuator force vector to a resulting force vector (*note that these vectors include force and torque elements*). Since we considered an underwater robot equipped with thrusters, the mapping is from a thruster force vector ($F_{m}$ space) to a body-frame vector ($F_{B}$ space) (see Figure 3). The mapping operator is a matrix, which has different names in the literature such as: control effectiveness matrix [4,17], static transformation matrix [18], geometrical distribution of thrusters [19], configuration matrix [16]. In this paper, the mapping of an actuator configuration is called a *configuration matrix*, denoted as A.

The paper is organized as follows. The notations are given in Section 2. The problem formulation and performance indices are described in Section 3. The problem’s solution is displayed in Section 4. Simulation results and analyses are depicted in Section 5. Real experiments are depicted in Section 6. Finally, conclusions and future works are discussed in Section 7.

## 2. Notation

**Figure 4:**
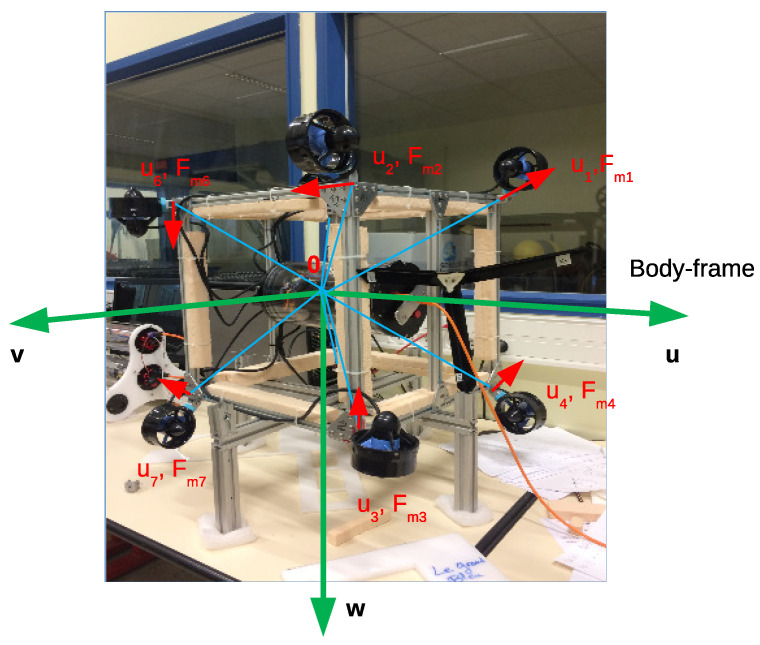
A given robot configuration.

This section provides most of notations used in the whole paper. However, further notations are introduced when needed. In order to illustrate the notations, a given robot configuration is shown in Figure 4, and detailed explanations are given in Table 1.

## 3. Problem Formulation

The relation between the desired force ($F_{B}^{d}$) and resulting force ($F_{B}$) depends on different elements (see Figure 2). This paper only focuses on the actuator configuration. Therefore, three assumptions were considered:

1. *The inverse characteristics and direct characteristics of the actuators are perfectly known, i.e., $F_{m}^{d}=F_{m}$*;
2. *The dispatcher is the Moore–Penrose pseudo-inverse of the actuators’ configuration, i.e., if the actuators’ configuration is the A matrix, the dispatcher is $D=A^{+}$;*
3. *All actuators have the same characteristics.*

### 3.1. Model of the Actuator Configuration

**Figure 5:**
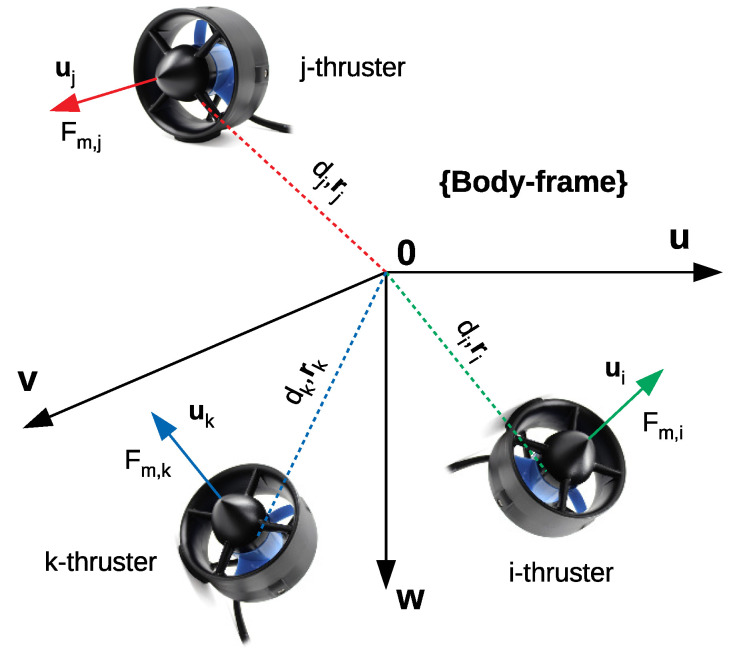
Actuator configuration model.

This part describes how to model an actuator configuration of an overactuated underwater robot equipped with thrusters. A thruster is modeled by the position and direction of the force produced with respect to the body-frame of the robot. The position of the *i*th thruster is described by a unit position vector $r_{i}$ and the distance $d_{i}$ to the system’s Center of Mass (CM) in the body-frame. The direction of the *i*th thruster is represented by a unit direction vector $u_{i}$ with respect to the body-frame as in Figure 5, and the *i*th thruster induces a force with the magnitude denoted as $F_{m,i}$. The relation of the thruster force vector and resulting force vector (note that this space includes force elements (F) and torque elements (Γ)) is described in Equation (1).

(1) FB=AFm=FΓ

where $F_{B}={\left[F_{u}\;F_{v}\;F_{w}\;F_{p}\;F_{q}\;F_{r}\right]}^{T}\in R^{6}$, $A\in R^{6\times m}$, $F_{m}={\left[F_{m,1}\;F_{m,2}\ldots F_{m,m}\right]}^{T}$$\in R^{m}$, and *m* is the number of thrusters, m>6. The configuration matrix A is described as:

(2) $$\begin{array}{c} \begin{array}{cc} A & =\left(\begin{array}{cccc} u_{1} & u_{2} & \cdots & u_{m}\\ d_{1}r_{1}⊗u_{1} & d_{2}r_{2}⊗u_{2} & \cdots & d_{m}r_{m}⊗u_{m} \end{array}\right)\\ \; & =\left(\begin{array}{cccc} u_{1} & u_{2} & \cdots & u_{m}\\ \tau_{1} & \tau_{2} & \cdots & \tau_{m} \end{array}\right)=\left(\begin{array}{c} A_{1}\\ A_{2} \end{array}\right) \end{array} \end{array}$$

where $A_{1}$ and $A_{2}\in R^{3\times m}$ are submatrices of A, which correspond to the force and torque elements, respectively. It is obvious to see that $\tau_{i}^{T}.u_{i}=0$. This is one constraint of the configuration matrix.

In this paper, we assumed that all distances from the thruster positions to the center of the body-frame are the same, $d_{i}=d_{j}=const,i,j=1,\ldots ,m$. Without loss of generality, we can assume that $d_{i}=1,i=1,\ldots ,m$.

### 3.2. Manipulability Index

**Figure 6:**
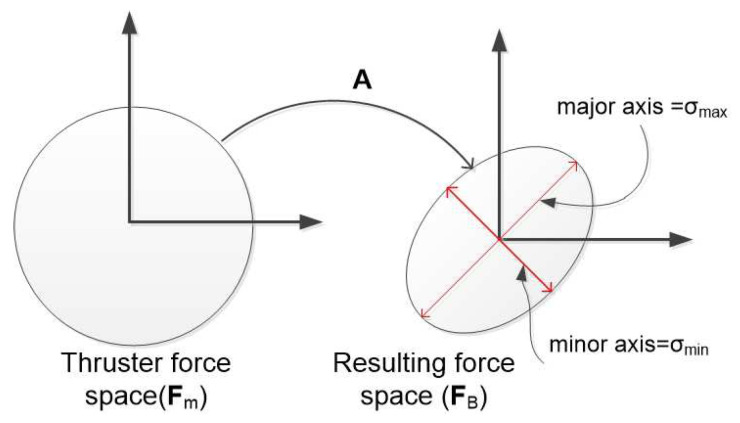
Manipulability ellipsoid with mapping.

As mentioned before, the manipulability index was first introduced in [20] for manipulator mechanisms, and there are different ways to quantify the manipulability index. This paper focuses on the isotropic property of a marine robot. Then, the ratio between the maximum and minimum radii of the manipulability ellipsoid was chosen (see Figure 6). Because of the units’ consistency, the matrices that relate to the force space, $A_{1}$, and torque space, $A_{2}$, were investigated separately. However, because of our assumption on $d_{i}$, the manipulability index is defined as the condition number of the configuration matrix:

(3) $$\begin{array}{cc} I_{m} & =Cond\left(A\right)=\frac{\sigma_{max}}{\sigma_{min}} \end{array}$$

where $\sigma_{max}$ and $\sigma_{min}$ are the maximum and minimum singular values of the configuration matrix, A, respectively.

Following Figure 6, the manipulability index investigates the resulting force ellipsoid, which is realizable by the thruster forces ($F_{m}$) such that $∥F_{m}∥\leq 1$ (see Theorem in Appendix A). If $I_{m}=1$, the robot is isotropic, or if $I_{m}=\infty$, the robot cannot act along at least one direction.

### 3.3. Energetic Index

Energy is very important for marine robots, and the energy consumption of robots depends on many factors such as the mechanical design, the environmental effects, and the specific mission. In order to evaluate the energy performance of an underwater robot, the energetic index was introduced in [15]. Being different from this, in our paper, the two-norm of the thruster force vector, $p_{E}={∥F_{m}∥}_{2}$, was used to quantify the energy that an underwater robot consumes to produce forces and torques, which can be calculated as follows in Equation (4):

(4) $$\begin{array}{cc} p_{E}={∥F_{m}∥}_{2} & =\sqrt{\sum_{i=1}^{m}F_{mi}^{2}}={∥A^{+}.F_{B}^{d}∥}_{2} \end{array}$$

**Figure 7:**
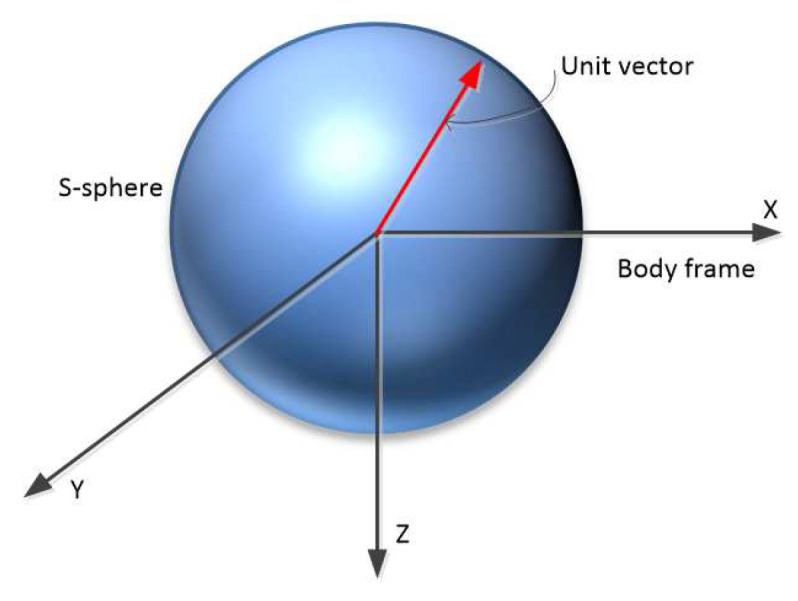
The rotation of the unit desired vector in the 3D sphere.

The energetic index is proposed to measure the variation of the energy consumption of an underwater robot when the direction of the desired force changes. It is quantified by computing the energy consumption when a unit desired force vector, ($F_{B}^{d}$), changes over the unit hypersphere (see Figure 7 for a 3D sphere). Because of the units’ consistency, however, the force and torque sphere were computed separately.

For the force sphere case, the unit desired force vector includes a unit vector of force elements and a zero vector of torque elements. For the torque sphere case, the unit desired force vector includes a zero vector of force elements and a unit vector of torque elements. Intuitively, this can be expressed as:

(5) FBd=FΓ=us0,for the force sphere0us,for the torque sphere.

where $u_{s}={\left[\cos\alpha \cos\beta \;\sin\alpha \cos\beta \;\sin\beta \right]}^{T}$ is a unit vector in spherical coordinates with α∈[−π,π] and β∈[−π/2,π/2].

According to these two cases, the norm of the thruster force vector was also divided into two cases as follows:

(6) pE=pEf=∥A+us0∥,for the force sphere casepEΓ=∥A+0us∥,for the torque sphere case.

The energetic index is defined as:

(7) $$\begin{array}{cc} I_{e} & =\frac{1}{S}\int_{S}\left(w_{ef}p_{Ef}+w_{e\Gamma }p_{E\Gamma }\right)dS \end{array}$$

where *S* is the area of the three-dimensional sphere, $p_{Ef}$, $p_{E\Gamma }$ are the subvectors of $p_{E}$ corresponding to the force sphere and the torque sphere case, respectively, and $w_{ef}$ and $w_{e\Gamma }$ are the weighting coefficients. Note that the weighting coefficients were chosen to normalize the difference between the force and torque spheres. These choices depends on the robot’s characteristics. However, because of the normalization of the spheres, it is normal to assign one to these coefficients.

### 3.4. Workspace Index

**Figure 8:**
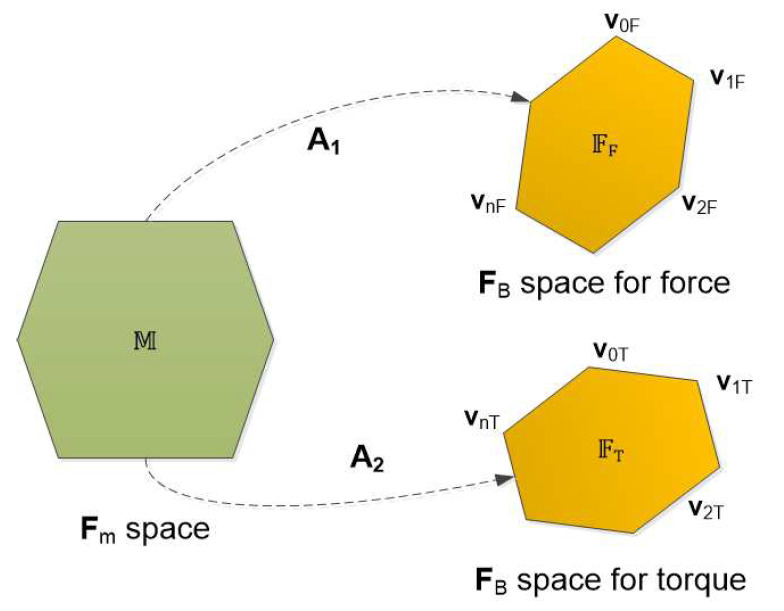
Space mapping ($v_{i}$ is denoted as the vertex).

The term workspace volume was first introduced in [13] for manipulator mechanisms. In this paper, the work space index was used to measure the volume of the attainable regions of the resulting force space with respect to (w.r.t.) the body-frame. In general, the characteristics of thrusters always have limitations, namely saturations and dead-zones (in this index, the dead-zone is not considered). This yields the polytope of the thruster force space, the $F_{m}$ space, denoted as M. By properly choosing the configuration matrix, $A={\left(A_{1}A_{2}\right)}^{T}$, the volume of the resulting force space for the force, the $F_{F}$ space, and the resulting force space for the torque, the $F_{T}$ space, can be maximized (see Figure 8). Note that the resulting spaces for the force and torque were studied separately because of the units’ consistency.

In general, the set M of thruster forces is known (with the given saturations of thrusters), so M is a polytope and $F_{F}$ and $F_{T}$ are also polytopes (under the $A_{1}$ and $A_{2}$ linear transform actions). We define the workspace index as:

(8) $$\begin{array}{cc} I_{w} & =\omega_{wf}Vol\left(F_{F}\right)+\omega_{w\tau }Vol\left(F_{T}\right) \end{array}$$

where Vol is the volume of a polytope and $\omega_{wf}$ and $\omega_{w\tau }$ are the weighting coefficients.

In control perspectives, the larger the space’s volumes are, the less control effort is need. The design objective was to maximize the workspace index, $I_{w}$. Normally, the set M is convex and its vertices are known. It is easy to find the vertices of $F_{F}$ and $F_{T}$. Of course, $F_{F}$ and $F_{T}$ are also convex sets (because of the linear transformation). This problem becomes a volume computation of convex polytopes.

### 3.5. Reactive Index

**Figure 9:**
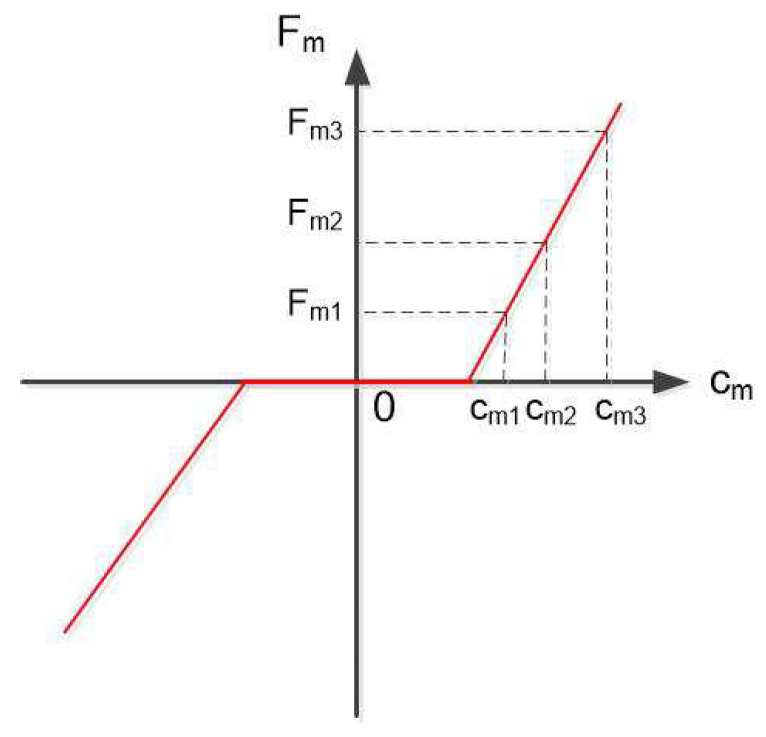
Thruster characteristics’ approximation.

The reactive index quantifies how fast the actuation system is able to change the orientation of the resulting force $F_{B}$ (ideally, $F_{B}^{d}$). Suppose that the robot is traveling in a direction with a set of thruster forces $F_{m1}$ induced from desired force vector $F_{B1}^{d}$. The robot wants to change to another direction (or the same direction with a different magnitude) with the desired force vector $F_{B2}^{d}$, so the thrusters have to produce another set of thruster forces $F_{m2}$. The two-norm of the deviation of the thruster forces, $▵F_{m}=F_{m1}-F_{m2}={\left[▵F_{m1}▵F_{m2}\cdot \cdot \cdot ▵F_{mm}\right]}^{T}$, is considered as the reactive capability of the robot. Referring to the approximation of the characteristics of the thrusters, as Figure 9, the change from $F_{m1}$ to $F_{m2}$ is closer than that from $F_{m1}$ to $F_{m3}$ (in the linear section, the dead-zone of the thruster characteristics is neglected in this paper). Hence, we have:

(9) $$\begin{array}{cc} ▵F_{m} & =A^{+}\left(F_{B1}^{d}-F_{B2}^{d}\right)=A^{+}▵F_{B}^{d} \end{array}$$

(10) $$\begin{array}{cc} ∥▵F_{m}∥ & =∥A^{+}▵F_{B}^{d}∥\leq ∥A^{+}∥∥▵F_{B}^{d}∥ \end{array}$$

(11) $$\begin{array}{c} \frac{∥▵F_{m}∥}{∥▵F_{B}^{d}∥}\leq ∥A^{+}∥ \end{array}$$

From Equation (11), the sensitivity of the thruster forces with respect to the desired forces, in other words the variation of the thruster forces w.r.t. the desired forces, is upper-bounded by the norm of the pseudo-inverse of the configuration matrix, $∥A^{+}∥$. We define the reactive index as:

(12) $$\begin{array}{cc} I_{re} & =∥A^{+}∥ \end{array}$$

It is obvious to see that if this index is lower, the robot is more reactive. Then, the objective of the design process is to minimize the reactive index.

### 3.6. Robustness Index

This criterion measures the robustness level of the AS of an underwater robot. This means that if any thruster of the robot fails, the remaining ones can still perform the robot’s mission. In particular, for any $F_{B}^{d}$ vector, there always exists a $F_{m}$ vector to satisfy the equation $F_{B}=AF_{m}$, and $F_{B}$ is as close as possible to $F_{B}^{d}$.

We have:

(13) $$\begin{array}{cc} F_{B} & =AF_{m}=\sum_{i=1}^{m}a_{i}F_{m,i} \end{array}$$

where $a_{i}$ is the *i*th column of the matrix A and $F_{m,i}$ is the force magnitude of the *i*th thruster.

When one or more thrusters completely fail, the value of $F_{m,i}=0$. Note that in the case that the *i*th thruster has partly failed, the value of $F_{m,i}$ remains small (not addressed in this paper). This is equivalent, as we considered that a corresponding column $a_{i}$ of the configuration matrix A equals the zero vector. Therefore, Equation (13) is equivalent to:

(14) $$\begin{array}{cc} F_{B} & =A^{\prime }F_{m} \end{array}$$

where the $A^{\prime }$ matrix is the A matrix with one or more corresponding columns equal to the zero vectors.

We discuss hereafter two questions: What are the conditions of the matrix $A^{\prime }$ to guarantee the robustness? What is the maximum number of failed thrusters?

To address these two questions, we supposed that *k* thrusters fail, and Equation (14) is a linear equation system with six equations (the dimensions of $F_{B}$ are 6×1) and (m−k) variables, because the matrix $A^{\prime }$ is 6×m, where *k* columns are zero vectors. It is obvious to see that if $rank(A^{\prime })=6$, for a given $F_{B}^{d}$, there always exits $F_{m}$ such that $F_{B}=A^{\prime }F_{m}$ and $F_{B}$ is as close as possible to $F_{B}^{d}$. This can be interpreted as m−k≥6 or k≤m−6. The condition on the configuration matrix and that on the maximum number of failed thruster that guarantee the robustness of an underwater robot are stated as:

1. *The maximum failed thrusters: m−6;*
2. *Robustness condition: the rank of the configuration matrix always equals six, i.e., rank(A)=6, if any columns, from one to a maximum of (m−6), of the A matrix equal the zero vectors. If rank(A)<6, the system becomes underactuated, and the guidance and control have to change to guarantee the robot’s mission. This problem is not addressed in this paper.*

We define the robustness index as:

(15) $$\begin{array}{cc} I_{ro} & =rank(A{|}_{\leq m-6})=6 \end{array}$$

where ${A|}_{\leq m-6}$ is the A matrix where the maximum number of columns being zero is (m−6). This index is verified in the solving process of the problem.

### 3.7. Configuration Matrix Design Problem

With all the performance indices discussed above, we propose the following design problem:

(16) $$\begin{array}{cc} \min_{A} & V\left(A\right)=\min_{A}{\left[I_{m}\;I_{e}\;\frac{1}{I_{w}}\;I_{re}\right]}^{T}\\ s.t & \;A\in A \end{array}$$

where V(A) is the objective function vector. A is the feasible set of the configuration matrix (A) including the constraints of the configuration matrix (A) and the robustness index. The reciprocal of the workspace index, $\frac{1}{I_{w}}$, is in Equation (16), because we wanted to maximize the workspace index.

This is a multi-objective optimization problem, and the unique solution belongs to the convexity of each objective function in the objective vector and the feasible set, A. Note that this optimization problem is with respect to a matrix variable (*matrix optimization*), not a vector variable. However, the optimization techniques for vector variables (*vector optimization*) can be applied here because we do not lose the physical meaning when converting a matrix variable to a vector variable in this optimization problem (because of the independency of each column in the matrix derived from the independent positions and orientations of the thrusters).

Specifically, Equation (16) can be rewritten as:

(17) $$\begin{array}{cc} \min_{A} & V\left(A\right)=\min_{A}{\left[I_{m}\;I_{e}\;\frac{1}{I_{w}}\;I_{re}\right]}^{T}\\ s.t\; & ∥u_{i}∥=1,i=1,2,\ldots ,m\\ \; & ∥\tau_{i}∥\leq 1,i=1,2,\ldots ,m\\ \; & \tau_{i}^{T}u_{i}=0,i=1,2,\ldots ,m\\ \; & I_{ro}=rank\left(A{|}_{\leq m-6}\right)=6 \end{array}$$

The problem (17) is to minimize an objective vector V(A), including the manipulability index, the energetic index, the reciprocal of the workspace index, and the reactive index, with respect to configuration matrix, A, and to satisfy the constraints of the matrix structure itself and the robustness index. It is clear that this is a nonconvex and multi-objective optimization problem, which normally has many solutions. In the following sections, we propose a mathematical analysis and a method for solving the multi-objective optimization problem.

## 4. Problem Solution

Our final objective was to find a distribution (position and orientation) of the thrusters of an underwater robot. This means obtaining the $u_{i}$ and $r_{i}$ vectors for i=1,2,…,m. These vectors can be extracted from configuration matrix A, which is the solution of the problem (17). Recall that our problem (17) is the multi-objective optimization problem with nonconvexity, and theoretically, this problem has infinitely many Pareto-optimal solutions. Our objective was to find one Pareto-optimal solution to build the robot. Analyzing the underlying mathematical properties of the problem helped us simplify the solving process. Thus, the mathematical analysis of the problem is shown in the next section.

### 4.1. Mathematical Analysis

The configuration matrix A has the form:

(18) $$A\begin{array}{c} =\left(\begin{array}{cccc} u_{1} & u_{2} & \cdots & u_{m}\\ \tau_{1} & \tau_{2} & \cdots & \tau_{m} \end{array}\right) \end{array}$$

We have:

(19) $$B=A^{T}A\begin{array}{c} \begin{array}{c} ={\left(\begin{array}{cccc} u_{1} & u_{2} & \cdots & u_{m}\\ \tau_{1} & \tau_{2} & \cdots & \tau_{m} \end{array}\right)}^{T}\left(\begin{array}{cccc} u_{1} & u_{2} & \cdots & u_{m}\\ \tau_{1} & \tau_{2} & \cdots & \tau_{m} \end{array}\right) \end{array} \end{array}$$

B is an m×m symmetric matrix where each element is denoted as $b_{ij}$. We have:

(20) $$\begin{array}{cc} Tr(B) & =\sum_{i=1}^{m}b_{ii}\\ \; & =\sum_{i=1}^{m}\lambda_{i} \end{array}$$

where $\lambda_{i}$ is the *i*th eigenvalue of matrix B.

From Equations (19) and (20), we have:

(21) $$\begin{array}{cc} \sum_{i=1}^{m}\lambda_{i} & =\sum_{i=1}^{m}u_{i}^{T}u_{i}+\tau_{i}^{T}\tau_{i}\\ \; & =\sum_{i=1}^{m}∥u_{i}{∥}^{2}+{∥\tau_{i}∥}^{2}\\ \sum_{i=1}^{m}\lambda_{i} & =\sum_{i=1}^{m}(1+∥\tau_{i}{∥}^{2}) \end{array}$$

In the case of manipulability index optimization, the condition of configuration matrix A is one, cond(A)=1. This means that the maximum singular value equals the minimum singular value, $\sigma_{max}=\sigma_{min}$. Note that the matrix A is the n×m matrix with n<m. The matrix A has *n* nonzero singular values (we have to guarantee that rank(A)=n), then the matrix B has *n* nonzero eigenvalues and m−n zero eigenvalues.

In the optimization case of the manipulability index, $cond\left(A\right)=1\Rightarrow \sigma_{max}=\sigma_{min}$, we have $\lambda_{i}=\lambda_{max}=\lambda_{min}=\lambda$ ($\sigma =\sqrt{\lambda }$). Equation (21) is rewritten as:

(22) $$\begin{array}{cc} n\lambda & =m+\sum_{i=1}^{m}{∥\tau_{i}∥}^{2}\\ \lambda & =\frac{m}{n}+\frac{1}{n}\sum_{i=1}^{m}{∥\tau_{i}∥}^{2} \end{array}$$

Considering the fact that $∥\tau_{i}{∥}^{2}\leq 1$, we have:

(23) $$\begin{array}{cc} \lambda & \leq 2.\frac{m}{n} \end{array}$$

Therefore, we have $\lambda_{max}=2\frac{m}{n}$ when $∥\tau_{i}{∥}^{2}=1$.

In the singular-value decomposition of a matrix, when cond(A)=1, the matrix A can be written as:

(24) $$\begin{array}{cc} A & =USV^{T}=U{\left[\sigma \right]}_{n\times m}V^{T} \end{array}$$

where $U\in R^{n\times n}$, $V\in R^{m\times m}$ are orthogonal matrices, $S={\left[\sigma \right]}_{n\times m}=\left(\begin{array}{cccc} \sigma & 0 & \cdots & 0\\ \vdots & \sigma & \cdots & 0\\ 0 & \cdots & \sigma & 0 \end{array}\right)\in R^{n\times m}$

The pseudo-inverse of matrix A is $A^{+}$ and can be written as:

(25) $$\begin{array}{cc} A^{+} & =VS^{+}U^{T}=V{\left[\frac{1}{\sigma }\right]}_{m\times n}U^{T} \end{array}$$

where $S^{+}={\left[\frac{1}{\sigma }\right]}_{m\times n}=\left(\begin{array}{ccc} \frac{1}{\sigma } & \cdots & 0\\ \vdots & \frac{1}{\sigma } & 0\\ 0 & 0 & \frac{1}{\sigma }\\ 0 & \cdots & 0 \end{array}\right)\in R^{m\times n}$

Our objective for the reactive index was to minimize $∥A^{+}∥$. From Equation (25), the reactive index $I_{re}=∥A^{+}∥=\frac{1}{\sigma }$, and the minimum value of the reactive index is equivalent to the maximum value of σ. This leads to the equality of Equation (23).

In order to minimize the reactive index and the manipulability index, the configuration matrix A has the following structure:

(26) $$\begin{array}{cc} A & =USV^{T}\\ \; & =U\left(\begin{array}{cccccc} \sigma & 0 & \cdots & 0 & 0 & 0\\ 0 & \sigma & 0 & \cdots & 0 & 0\\ 0 & 0 & \sigma & 0 & \cdots & 0\\ \vdots & \vdots & \vdots & \vdots & \vdots & \vdots \\ 0 & 0 & 0 & \sigma & 0 & 0 \end{array}\right)V^{T} \end{array}$$

where S(n×m) is like-diagonal and $\sigma =\sqrt{\lambda }=\sqrt{2\frac{m}{n}}$; U(n×n) and V(m×m) are orthogonal matrices ($UU^{T}=I,VV^{T}=I$). This result can be used as the initial value of the numerical optimization process and is useful for solving the problem.

We continue to discuss the energetic index. First, we introduce a proposition as follows:

**Proposition** **1.** *Let M be a p×q matrix (p≥q), $M\in R^{p\times q}$. For all $x\in R^{q}$, if $M=P\Sigma Q^{T}$, where $P\in R^{p\times p},Q\in R^{q\times q}$ are orthogonal matrices, $\Sigma =\left(\begin{array}{cccc} \mu & 0 & \cdots & 0\\ 0 & \mu & \cdots & 0\\ 0 & \cdots & \mu & 0\\ 0 & \cdots & 0 & \mu \\ \vdots & \vdots & \vdots & \vdots \\ 0 & 0 & 0 & 0 \end{array}\right)\in R^{p\times q}$, then ∥Mx∥=∥M∥∥x∥.*

**Proof.**  We have:

(27) $$\begin{array}{cc} {∥Mx∥}^{2} & ={\left(Mx\right)}^{T}\left(Mx\right)=x^{T}M^{T}Mx \end{array}$$

With $M=P\Sigma Q^{T}$:

(28) $$\begin{array}{cc} {∥Mx∥}^{2} & =x^{T}{\left(P\Sigma Q^{T}\right)}^{T}\left(P\Sigma Q^{T}\right)x\\ \; & =x^{T}Q\Sigma^{T}P^{T}P\Sigma Q^{T}x\\ \; & =x^{T}Q\Sigma^{T}\Sigma Q^{T}x \end{array}$$

We have:

(29) $$\begin{array}{cc} \Sigma^{T}\Sigma & ={\left(\begin{array}{cccc} \mu & 0 & \cdots & 0\\ 0 & \mu & \cdots & 0\\ 0 & \cdots & \mu & 0\\ 0 & \cdots & 0 & \mu \\ \vdots & \vdots & \vdots & \vdots \\ 0 & 0 & 0 & 0 \end{array}\right)}^{T}\left(\begin{array}{cccc} \mu & 0 & \cdots & 0\\ 0 & \mu & \cdots & 0\\ 0 & \cdots & \mu & 0\\ 0 & \cdots & 0 & \mu \\ \vdots & \vdots & \vdots & \vdots \\ 0 & 0 & 0 & 0 \end{array}\right)\\ \; & =\left(\begin{array}{cccc} \mu^{2} & 0 & \cdots & 0\\ 0 & \mu^{2} & \cdots & 0\\ \vdots & \vdots & \vdots & \vdots \\ 0 & \cdots & 0 & \mu^{2} \end{array}\right)=\mu^{2}I \end{array}$$

where I is a q×q identity matrix.

Replacing Equation (29) to (28), we have:

(30) $$\begin{array}{cc} {∥Mx∥}^{2} & =x^{T}V\mu^{2}IV^{T}x\\ \; & =\mu^{2}x^{T}x={∥M∥}^{2}{∥x∥}^{2} \end{array}$$

Therefore, ∥Mx∥=∥M∥∥x∥.   □

The energetic index is stated as:

(31) $$\begin{array}{cc} I_{e} & =\frac{1}{S}\int_{S}(w_{ef}∥A^{+}(F_{B}^{d}\left(f\right)∥+w_{e\Gamma }∥A^{+}F_{B}^{d}\left(\Gamma \right)∥)dS \end{array}$$

Choosing $w_{ef}=w_{e\Gamma }=1$ (because the desired force vectors, $F_{B}^{d}\left(f\right),F_{B}^{d}\left(\tau \right)$, are units), we have:

(32) $$\begin{array}{cc} I_{e} & =\frac{1}{S}\int_{S}(∥A^{+}F_{B}^{d}\left(f\right)∥+∥A^{+}F_{B}^{d}\left(\Gamma \right)∥)dS \end{array}$$

In the case in which the reactive index and the manipulability index are minimum, the configuration matrix A(n×m) has the form of Equation (26); therefore, the pseudo-inverse matrix $A^{+}\left(m\times n,\;m>n\right)$ has the following structure:

(33) $$\begin{array}{cc} A^{+} & =VS^{+}U^{T}=V\left(\begin{array}{cccc} \frac{1}{\sigma } & 0 & \cdots & 0\\ 0 & \frac{1}{\sigma } & \cdots & 0\\ 0 & \cdots & \frac{1}{\sigma } & 0\\ 0 & \cdots & 0 & \frac{1}{\sigma }\\ \vdots & \vdots & \vdots & \vdots \\ 0 & 0 & 0 & 0 \end{array}\right)U^{T} \end{array}$$

where V,U are orthogonal matrices.

It is clear that matrix $A^{+}$ satisfies the condition of Proposition 1. Applying this proposition, we have: $∥A^{+}F_{B}^{d}\left(f\right)∥=∥A^{+}∥∥F_{B}^{d}\left(f\right)∥$ and $∥A^{+}F_{B}^{d}\left(\Gamma \right)∥=∥A^{+}∥∥F_{B}^{d}\left(\Gamma \right)∥$. Therefore, Equation (32) becomes:

(34) $$\begin{array}{cc} I_{e} & =\frac{1}{S}\int_{S}(∥A^{+}∥∥F_{B}^{d}\left(f\right)∥+∥A^{+}∥∥F_{B}^{d}\left(\Gamma \right)∥)dS\\ \; & =\frac{1}{S}∥A^{+}∥\int_{S}(∥F_{B}^{d}\left(f\right)∥+∥F_{B}^{d}\left(\Gamma \right)∥)dS\\ \; & =2∥A^{+}∥ \end{array}$$

From the aforementioned mathematical analysis of the energetic index, we can see that the energetic index belongs to the norm of the pseudo-inverse of the configuration matrix, $I_{re}=2∥A^{+}∥$, when the configuration matrix A has the form of (26).

We then discuss the upper-bound of the workspace index. For the units’ consistency, the workspace index for the force space and that for the torque space were investigated separately, denoted as $I_{wf}$ and $I_{w\tau }$, respectively. Recall that the objective of the workspace index is to maximize the volume of the resulting force space ($F_{B}$ space), including the resulting space for the force and the resulting space for the torque with given thrusters’ force (the $F_{m}$ space).

The fact that for all vectors $F_{m}\in R^{m}$, $∥AF_{m}∥\leq ∥A∥∥F_{m}∥$. The volume of the resulting force space is maximum when the equality holds.

**Figure 10:**
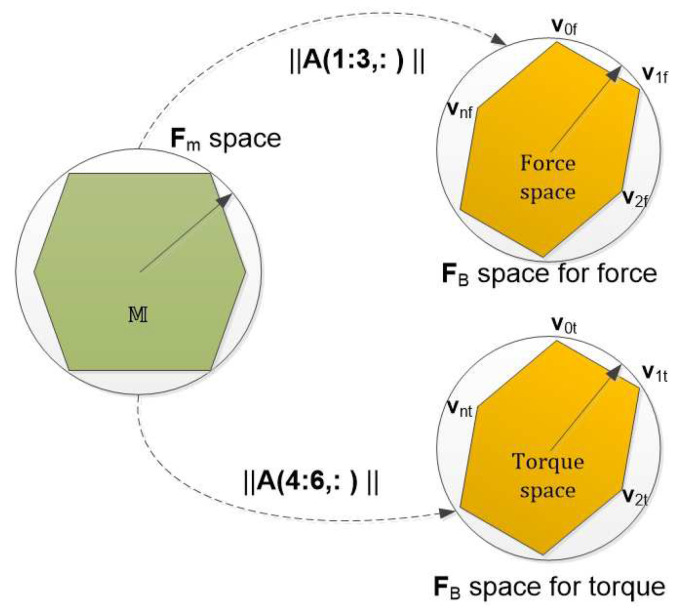
Upper-bound of the resulting force space.

Following Figure 10, the volumes of the resulting force spaces ($F_{B}$) (the force and torque spaces) are always less than the volumes of the exterior hypersphere of $F_{B}$ spaces of the force and torque (this may be the circumscribed spheres or not). This means that:

(35) $$\begin{array}{cc} I_{wF} & \leq Vol(B(R1))\\ I_{wT} & \leq Vol(B(R2)) \end{array}$$

where B(R1) and B(R2) are Euclidean balls of radius $R1=∥A\left(1:3,:\right)∥∥F_{m}∥=∥A_{1}∥∥F_{m}∥$ and of radius $R2=∥A\left(4:6,:\right)∥∥F_{m}∥=∥A_{2}∥∥F_{m}∥$, respectively; A(1:3,:) is composed of the first three rows of A, and A(4:6,:) is composed of the last three rows of A.

The volume of a Euclidean ball of radius *R* in *n*-dimensional Euclidean space is [21]:

(36) $$\begin{array}{cc} V_{n}\left(R\right) & =\left\{\begin{array}{cc} \frac{\pi^{k}}{k!}R^{2k}, & \mathrm{if}n=2k\\ \frac{2^{k+1}\pi^{k}}{(2k+1)!!}R^{2k+1}, & \mathrm{if}n=2k+1. \end{array}\right. \end{array}$$

where (2k+1)!!=1.3.5,…,(2k−1).(2k+1).

**Proposition** **2.** *If the configuration matrix A has the form of (26), then $cond\left(A_{1}\right)=cond\left(A_{2}\right)=1$ and $∥A_{1}∥=∥A_{2}∥=\sigma$.*

**Proof.**  We have:

(37) $$\begin{array}{cc} AA^{T} & =\left(USV^{T}\right){\left(USV^{T}\right)}^{T}=USV^{T}VS^{T}U^{T}\\ \; & =USS^{T}U^{T}=\sigma^{2}I \end{array}$$

On the other hand:

(38) AAT=A1A2A1A2T=A1A2(A1TA2T)=A1A1T00A2A2T

From (37) and (38), we have:

(39) $$\begin{array}{cc} A_{1}A_{1}^{T} & =\sigma^{2}I_{1}\\ A_{2}A_{2}^{T} & =\sigma^{2}I_{2} \end{array}$$

where $I_{1}$ and $I_{2}$ are partitioned matrices of matrix I.

From (39) and the uniqueness of singular-value decomposition [22], it is obvious to see that the structures of $A_{1}$ and $A_{2}$ are the same as (26) with different dimensions. Therefore, $cond\left(A_{1}\right)=cond\left(A_{1}\right)=1$ and $∥A_{1}∥=∥A_{2}∥=\sigma$.   □

From (35) and (36) and Proposition 2, it is obvious to obtain the upper-bound of the resulting spaces of the force and torque of the system and then the upper-bound of the workspace index. Normally, the weighting coefficients in the workspace index are chosen as one because of our assumption of $d_{i}$.

### 4.2. Problem Solution

Based on the above mathematical analysis, the goal-attainment method was chosen to solve the problem with the given desired values. The idea of this method is to minimize the deviation of the desired values and the obtained values. One advantage of the goal-attainment method is that the problem does not need to be normalized to a dimensionless problem. The solution of this method has been proven to be Pareto-optimal. This method is also suitable when the feasible objective set is nonconvex [23]. All Pareto-optimal solutions may be found by changing the attainment vector; however, this depends on the properties of the problem.

**Figure 11:**
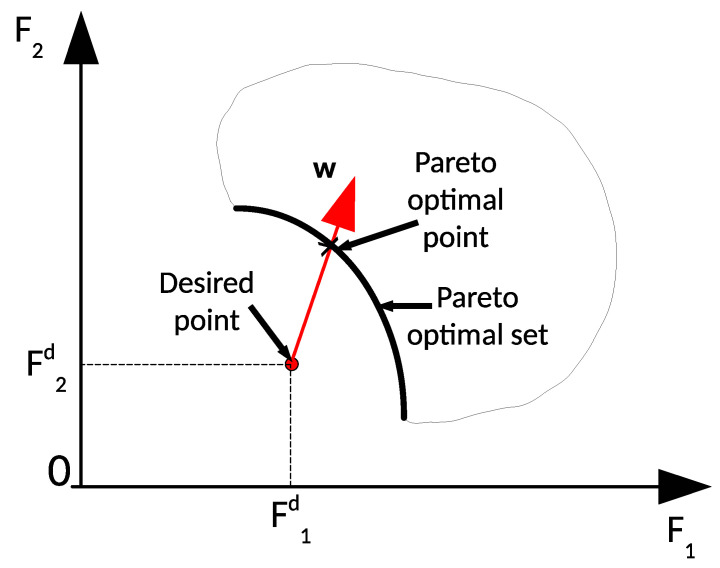
Goal-attainment method with two objective functions.

Our problem using the goal-attainment method becomes:

(40) $$\begin{array}{cc} \min_{A,\gamma } & \gamma \\ s.t & A\in \bar{A}\\ \; & V\left(A\right)-w\gamma \leq V_{goal} \end{array}$$

where $\bar{A}=A\backslash I_{ro}$, i.e., the feasible set of configuration matrices, A, without robustness index $I_{ro}$, γ is a slack vector variable, and $V_{goal}=\left[I_{m}^{d}\;I_{e}^{d}\;\frac{1}{I_{w}^{d}}\;I_{re}^{d}\right]$ is the desired objective vector, w is an attainment vector, which can be chosen. The goal-attainment method with two objective functions is illustrated in Figure 11. By altering w vector, we searched for the Pareto-optimal solutions.

Therefore, our solving process included two phases:

1. Phase 1: Find one Pareto-optimal solution of the configuration matrix with the goal-attainment method;
2. Phase 2: Check the robustness index of the chosen solution in Phase 1.

The optimization toolbox in the MATLAB environment was used to solve our problem. Note that our problem was formulated as a multi-objective optimization problem. One objective has one desired value excluding the robustness index, which is as a constraint, and therefore, the desired vector is set up. The goal-attainment method was used to solve the problem. An attainment vector was chosen as a trade-off between the underachievement and overachievement of the objective functions. In multi-objective optimization, an optimal solution depends on a decision maker. Theoretically, there is no method for this choice. In our work, this vector was selected by trial and error. In particular, for the manipulability, reactivity, and energetic indices, we know exactly the desired values, so the corresponding values in the attainment vector were chosen as zero, which means that these are hard constraints. For the workspace index, we only know the upper-bound of the desired value; therefore, a positive value was chosen for underachievement.

## 5. Simulation Results

We designed an overactuated underwater robot with m=8 thrusters and n=6 degrees of freedom. Two cases were simulated: the general case and the given position case. In the general case, we have to identify both the positions and orientations of eight thrusters optimizing the performance indices. In the given position case, the thrusters are installed at the corners of a cube, and we only have to determine the directions of the thrusters. In this simulation, the thruster characteristics were chosen as in [5], then the maximum and minimum values of the thrusters forces were $F_{imax}=1.1N$ and $F_{imin}=-0.4N$, respectively. The desired values of the performance indices were subsequently $I_{m}^{d}=1,I_{e}^{d}=1.2248,I_{wF}^{d}=597.7,I_{wT}^{d}\;=\;597.7,I_{re}^{d}\;=\;0.6124$ ($\sigma^{max}=\sqrt{2\frac{m}{n}}=1.6330$; see Table 2 for more details).

### 5.1. General Case

In this case, the robot is called a ball robot, and the positions and orientations of the thrusters are not known. The problem (40) is solved as follows.

#### 5.1.1. Phase 1

The optimization toolbox was used to solve the problem (40) with the desired goal vector, and the constraints were $V_{goal}=\left[I_{m}^{d}\;I_{e}^{d}\;\frac{1}{I_{w}^{d}}\;I_{re}^{d}\right]={\left[1\;1.2248\;0.0033\;0.6124\right]}^{T}$, the constraint set $\bar{A}=\{A\in R^{6\times 8}/∥u_{i}∥=1,∥\tau_{i}∥\leq 1,\tau_{i}^{T}u_{i}=0\}$, and the attainment vector $w={\left[0\;0\;0\;0.0036\right]}^{T}$. The attainment vector allows setting the overachievement or underachievement of the individual goals. At the moment, there is no general method to choose this attainment vector. It was chosen by trial and error. By our approach, we found that some values of this vector can be assigned zero (this imposes hard constraints), except the workspace index (because of the upper-bound value).

**Figure 12:**
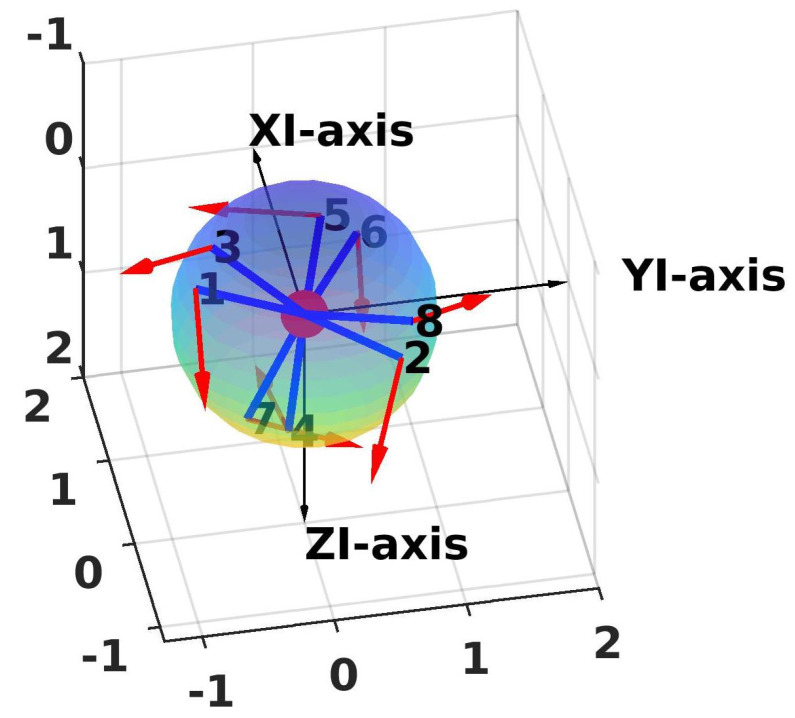
Positions and directions of the thrusters (general case) (XI-axis = u-axis; YI-axis = v-axis; ZI-axis=w-axis).

**Figure 13:**
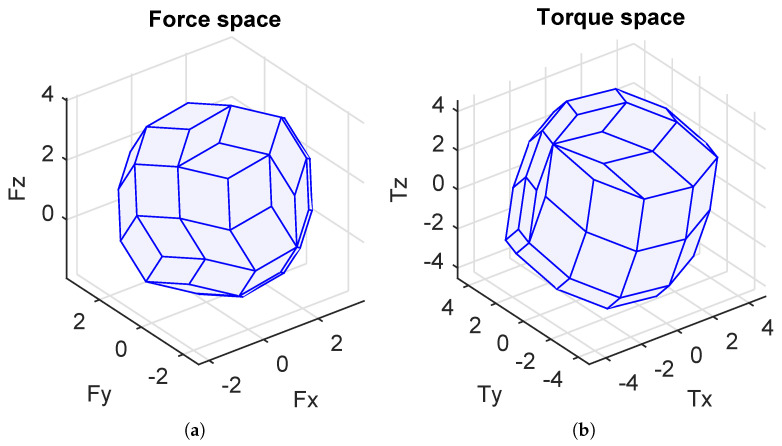
Attainable spaces in the general case (Fx-axis = u-axis; Fy-axis = v-axis; Fz-axis = w-axis). (**a**) Force space. (**b**) Torque space.

The simulation results are shown in Figure 12 and Figure 13a,b. The configuration matrix A and optimal values are shown in Table 3. Specifically, in Figure 12, the positions of the thrusters are at the top of the blue line, and the orientations of the thrusters are shown as the red arrow. Furthermore, we can see that the isotropic property of the robot is guaranteed (see Figure 13a,b) with the sphere shapes of the attainable spaces of the forces and torques. From Table 3, the obtained values of the manipulability index, the energetic index, and the reactive index were almost the same as the desired values. However, the obtained workspace index was smaller than the desired one.

#### 5.1.2. Phase 2

In this phase, the robustness index was checked. The optimal configuration matrix A in Table 3 satisfies the robustness constraint. Specifically, the maximum number of thrusters that are acceptable (for failures) is two.

### 5.2. Given Position Case

In this case, the robot is called the cube robot, and the positions of thrusters are at the corners of the cube. We only had to find their orientations. The number of variables in the problem (40) was reduced. The desired objective vector and attainment vector were the same as in general case. The results are presented in the sequel.

#### 5.2.1. Phase 1

**Figure 14:**
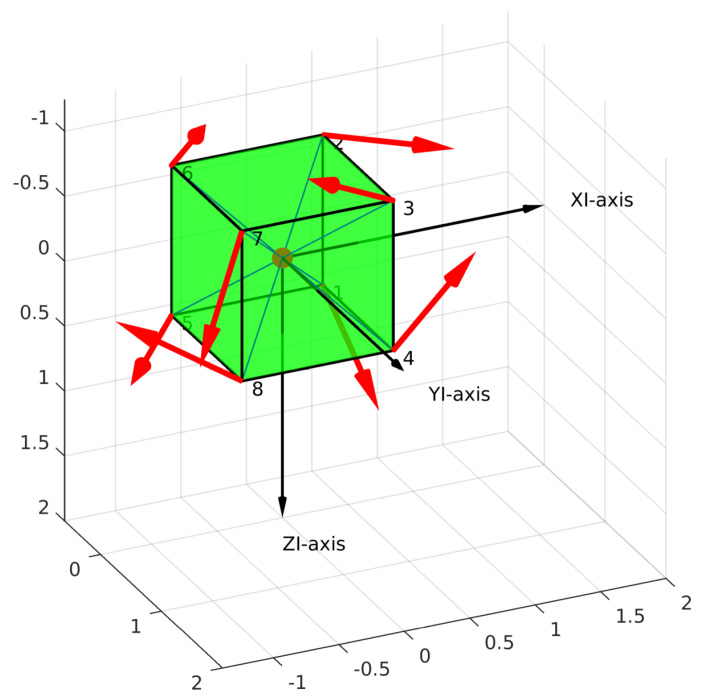
Robot design with the directions of the thrusters (given position case) (XI-axis = u-axis; YI-axis = v-axis; ZI-axis = w-axis).

**Figure 15:**
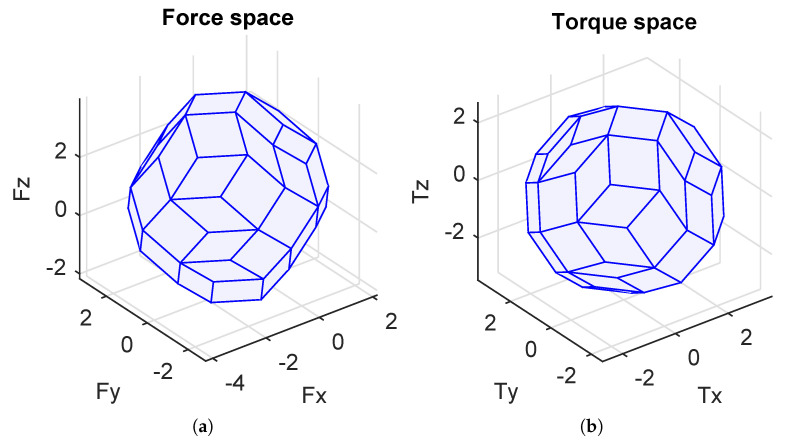
Attainable spaces in the given position case (Fx-axis = u-axis; Fy-axis = v-axis; Fz-axis = w-axis). (**a**) Force space. (**b**) Torque space.

The optimization toolbox was used to solve our problem, and the simulation results are shown in Figure 14 and Figure 15a,b and Table 4. The directions of the thrusters are depicted as red arrows in Figure 14. Being similar to the general case, the isotropic property is also guaranteed in this case (see Figure 15a,b). One Pareto-optimal configuration matrix is shown in Table 4. We can see that the obtained objective values in Table 4 are the same as the general case.

#### 5.2.2. Phase 2

The optimal configuration matrix A in Table 4 satisfies the conditions of the robustness index. Similarly, the maximum number of thrusters that can fail is two.

### 5.3. A Comparison of Two Configurations

In this section, a comparison of two configurations is illustrated. The choice of the configurations corresponds to a real robot (cube robot), which is used in the experiments in the next section. The first one is a normal configuration (denoted as $C^{1}$) in which the thrusters are distributed vertically or horizontally (in practice, this configuration is easier to install, as Figure 21 shows). The configuration matrix of the $C^{1}$ configuration, denoted as $A_{1}$, is shown in Equation (41).

(41) $$A_{1}=(\begin{array}{rrrrrrrr} 0 & 1 & 0 & 0 & 0 & 0 & -1 & 0\\ 1 & 0 & 0 & -1 & 1 & 0 & 0 & 0\\ 0 & 0 & -1 & 0 & 0 & 1 & 0 & -1\\ 0.27 & 0 & -0.27 & 0.27 & 0.27 & 0.27 & 0 & 0.27\\ 0 & -0.27 & 0.27 & 0 & 0 & 0.27 & -0.27 & -0.27\\ 0.27 & -0.27 & 0 & 0.27 & 0.27 & 0 & 0.27 & 0 \end{array})$$

**Figure 16:**
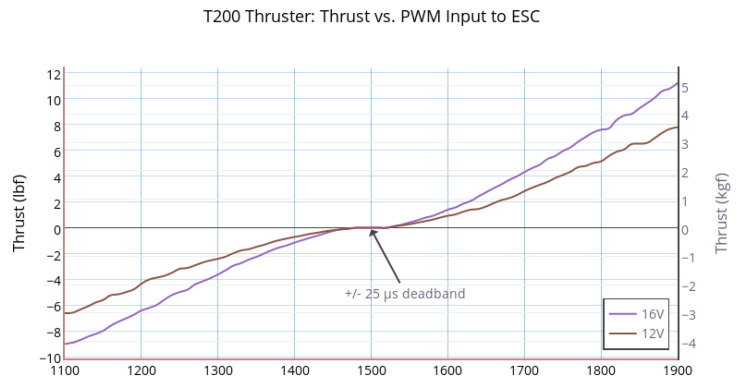
Thruster characteristics (BlueRobotics) [24].

The second one (denoted as $C^{2}$) is an optimal configuration, denoted as $A_{2}$, which is a solution of the optimization problem (given position case) thanks to the thruster characteristics of BlueRobotics (Figure 16), and the optimal configuration matrix is shown in Equation (42).

(42) $$\begin{array}{ccc} A_{2} & = & \left(\begin{array}{rrrrrrrr} 0.6616 & -0.8122 & 0.4785 & 0.0836 & -0.0836 & -0.4785 & -0.8122 & -0.6616\\ 0.7452 & 0.3337 & 0.3337 & 0.7452 & 0.7452 & 0.3337 & -0.3337 & 0.7452\\ -0.0836 & -0.4785 & -0.8122 & 0.6616 & -0.6616 & 0.8122 & -0.4785 & 0.0836\\ 0.1608 & 0.0111 & -0.2459 & -0.3708 & 0.3642 & 0.2015 & 0.0011 & -0.1658\\ -0.0989 & 0.3556 & 0.3633 & -0.0989 & -0.1056 & 0.3508 & -0.3456 & -0.1056\\ 0.3906 & 0.2292 & 0.0044 & 0.1583 & -0.1649 & -0.0254 & 0.2392 & -0.3708 \end{array}\right) \end{array}$$

**Figure 17:**
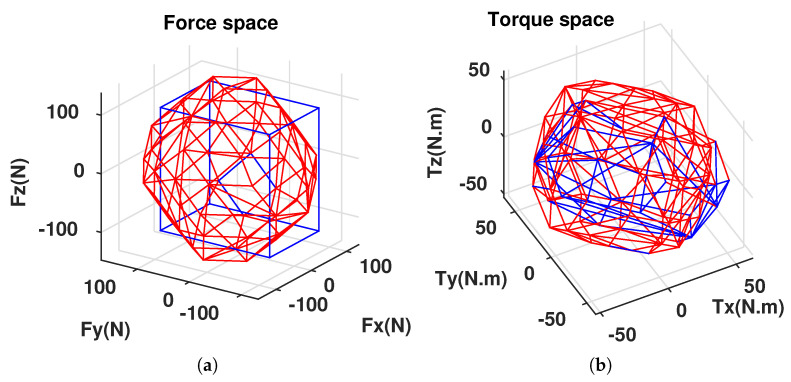
Attainable spaces for different configurations (X-axis = u-axis; Y-axis = v-axis; Z-axis = w-axis). (**a**) $C^{1}$(blue), $C^{2}$(red). (**b**) $C^{1}$(blue), $C^{2}$(red).

Note that the configuration matrices $A_{1}$ and $A_{2}$ were calibrated with the corresponding geometrical properties of the real cube robot at the LIRMM Institute, Montpellier University. The attainable force space and torque space corresponding to the two configurations $C^{1}$ and $C^{2}$ are illustrated in Figure 17a,b. It is obvious that the $C^{2}$ configuration is more isotropic than the $C^{1}$ configuration. However, for some specific points of the attainable force and torque spaces, the $C^{1}$ configuration is better than the $C^{2}$ configuration.

Thanks to the properties of matrices $A_{1}$ and $A_{2}$ (Equations (41) and (42)) and the thruster characteristics (Figure 16), Table 5 shows the values of the performance indices for both configurations. The performances of the $C^{2}$ configuration are better than $C^{1}$. Because of the calibration (the distance $d_{i}$ is different between the motors), the manipulability index ($I_{m}$) is larger than one (Note that, theoretically, the distances of all thrusters with respect to the center of mass were assumed the same (without loss of generality, they were assigned one). However, in practice, for our cube robot, these distances were not completely the same, and we had to calibrate the configuration matrix.).

**Figure 18:**
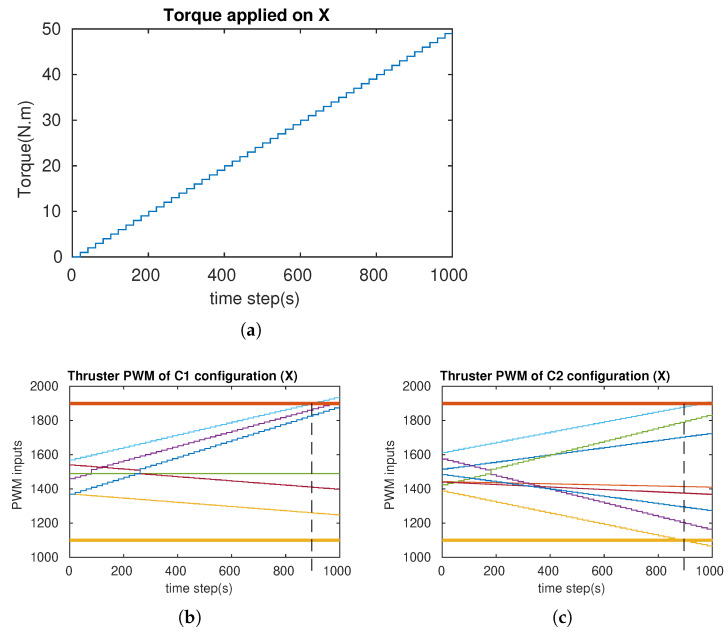
The simulation of the cube rotation about the u-axis for $C^{1}$ and $C^{2}$ (X-axis = u-axis). (**a**) Applied torque about the u-axis. (**b**) PWM inputs of $C^{1}$. (**c**) PWM inputs of $C^{2}$.

**Figure 19:**
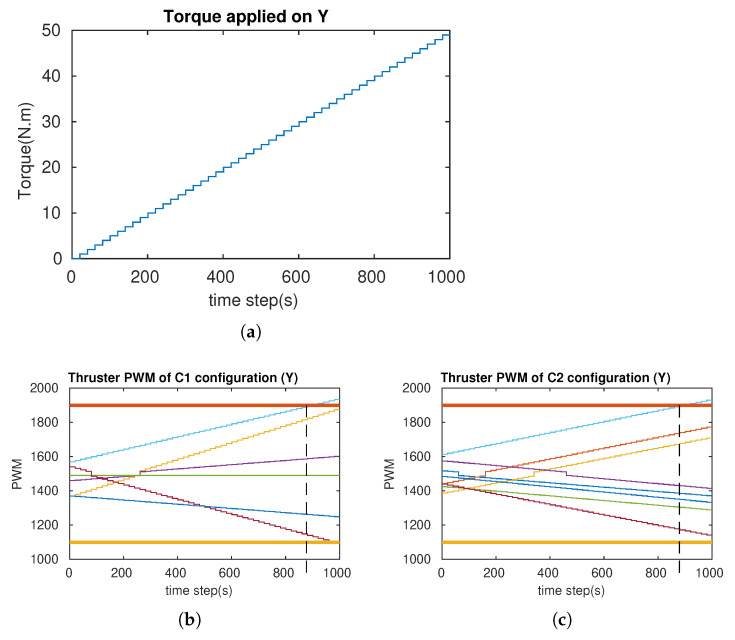
The simulation of the cube rotation about the v-axis for $C^{1}$ and $C^{2}$ (Y-axis = v-axis). (**a**) Applied torque about the v-axis. (**b**) PWM inputs of $C^{1}$. (**c**) PWM inputs of $C^{2}$.

**Figure 20:**
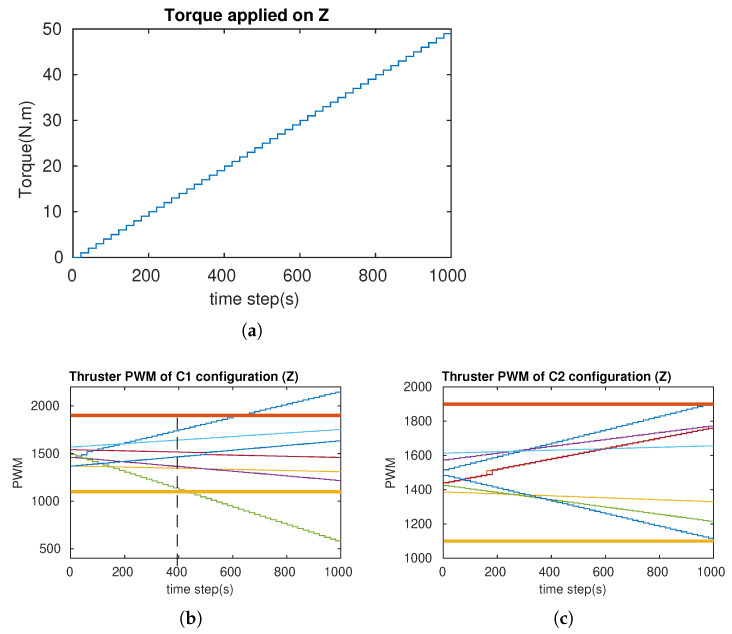
The simulation of the cube rotation about the Z-axis for $C^{1}$ and $C^{2}$ (Z-axis = w-axis). (**a**) Applied torque about the w-axis. (**b**) PWM inputs of $C^{1}$. (**c**) PWM inputs of $C^{2}$.

In order to verify the attainability of the two configurations (workspace index), incremental torques were applied about the u-, v-, and w-axis, respectively (Figure 18a, Figure 19a and Figure 20a), and the corresponding Pulse-Width Modulation (PWM) inputs ($c_{m}$) of the eight thrusters were computed. The results are shown in Figure 18b,c, Figure 19b,c and Figure 20b,c, in which the two PWM saturation values of the thrusters (upper saturation value: 1900, lower saturation value: 1100) are plotted with two bold lines. We can see that the performances of the robot with the two configurations are almost the same for the rotation about the u- and v-axis. However, the $C^{2}$ configuration showed better performance for the rotation about the w-axis. In fact, the thrusters with the $C^{1}$ configuration reached saturations very earlier in comparison with the thrusters with the $C^{2}$ configuration (Figure 20b,c).

In order to validate the robustness of the optimal configuration ($C^{2}$) in comparison with the normal configuration ($C^{1}$), the rank of matrices $A_{1}$ and $A_{2}$ was checked when one or two arbitrary columns have been nullified. When the resulting matrices are rank deficient, this means that the robustness is not guaranteed because one direction is not actuated. Therefore, we cannot control all 6 DoFs independently. The robustness index in Table 5 shows the checking results. In particular, when the fifth thruster of the $C^{1}$ configuration fails, the robustness is not guaranteed.

## 6. Experimental Results

**Figure 21:**
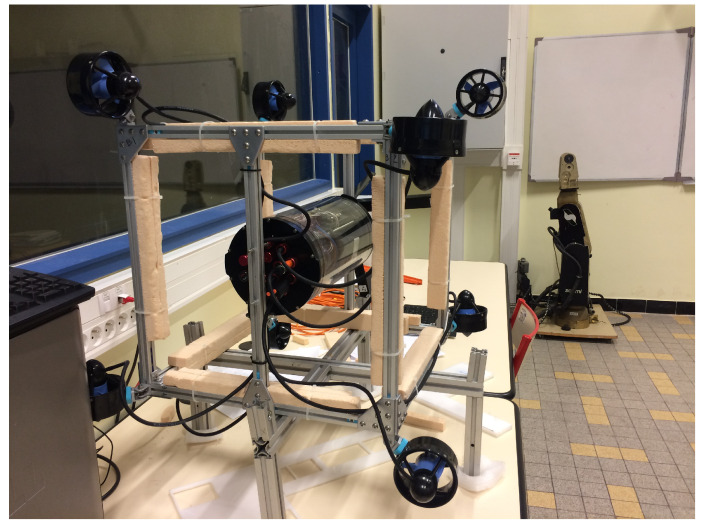
$C^{1}$ of the cube robot.

**Figure 22:**
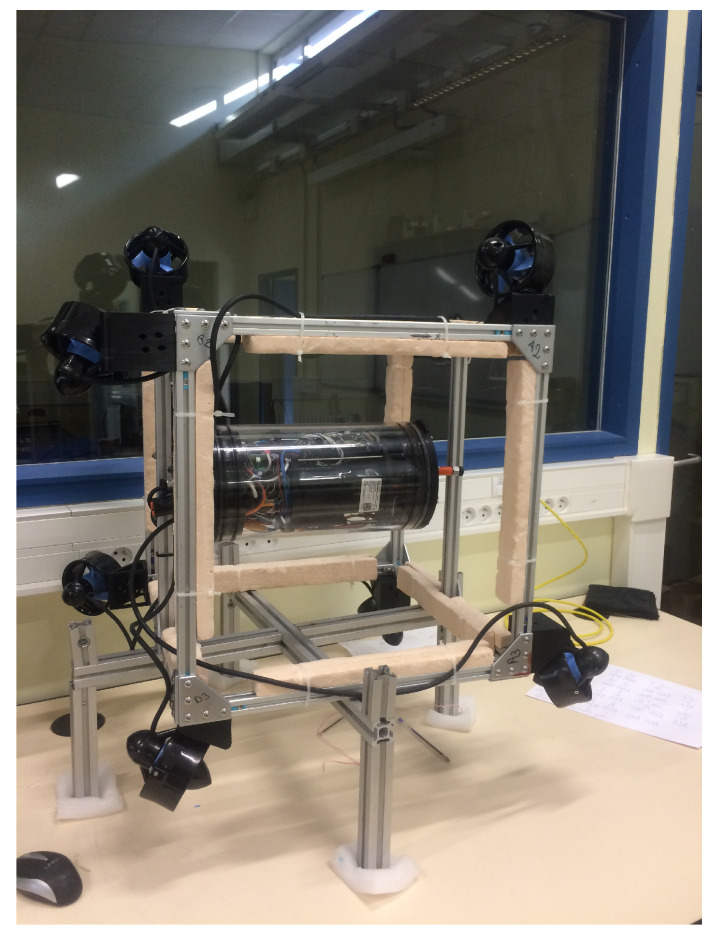
$C^{2}$ of the cube robot.

**Figure 23:**
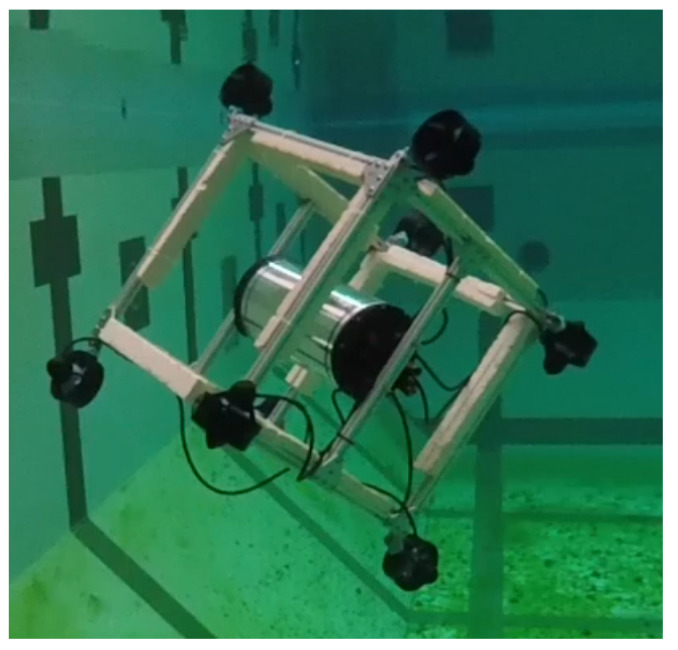
Cube robot in the water https://www.youtube.com/watch?v=RKiWUOxDKdw (accessed on 18 October 2019).

Experiments were carried out on the cube robot to compare between the two configurations, $C^{1}$ (see Figure 21) and $C^{2}$ (see Figure 22), in the swimming pool at Montpellier University. The cube in the water and a video link for the cube’s operations can be seen in Figure 23.

### 6.1. Attainability Validation

**Figure 24:**
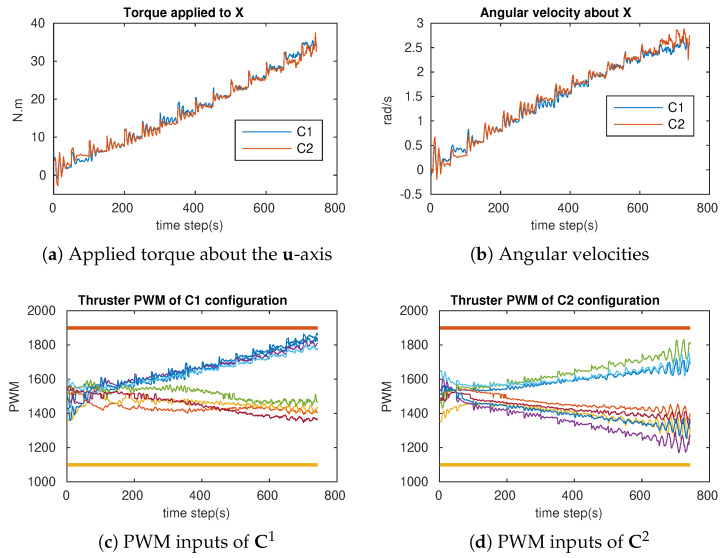
The cube rotates about the u-axis for $C^{1}$ and $C^{2}$ (X-axis = u-axis).

**Figure 25:**
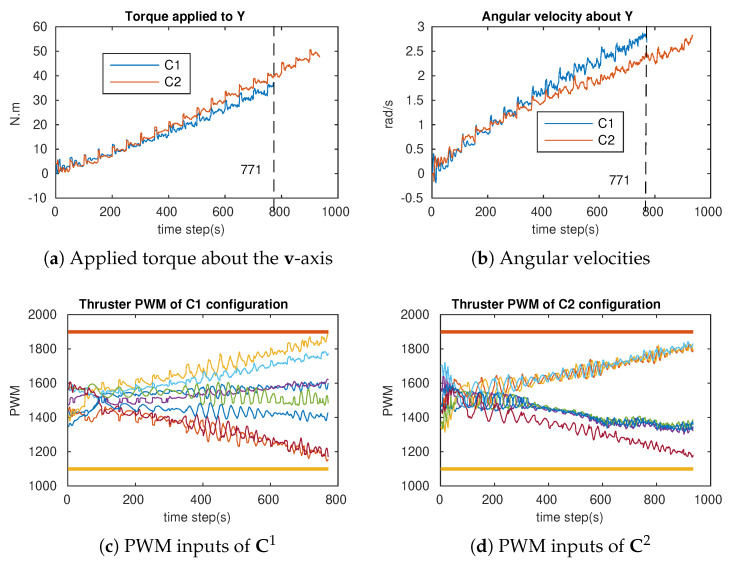
The cube rotates about the v-axis for $C^{1}$ and $C^{2}$ (Y-axis = v-axis).

**Figure 26:**
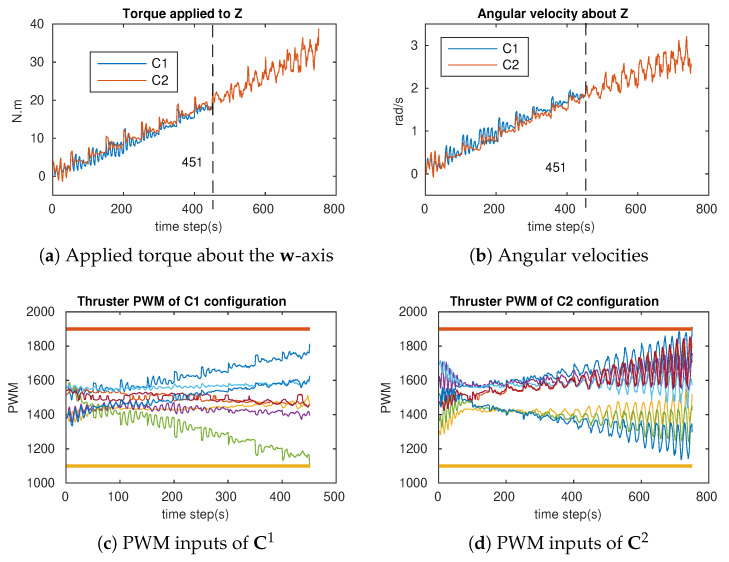
The cube rotates about the Z–axis for $C^{1}$ and $C^{2}$ (Z-axis = w-axis).

The incremental torques about the u-axis, v-axis, and w-axis were applied to cube robot respectively, and angular velocities and PWM input values were stored to evaluate these two configurations. For safety, the experiments were stopped when one thruster reached the saturation values. The experimental results are shown in Figure 24, Figure 25 and Figure 26. For rotating about the u-axis (Figure 24), the attainability of configurations $C^{1}$ and $C^{2}$ was almost the same: all thrusters operated in a feasible region. Otherwise, for rotating about the v-axis or w-axis, the attainability of configuration $C^{2}$ was better than that of $C^{1}$. In particular, with the v-axis experiment (Figure 25), the cube robot with $C^{1}$ stopped the mission earlier than with $C^{2}$ (at Time Step 771) because one thruster reached saturation. The same thing happened with the w-axis experiment (at Time Step 451) (see Figure 26).

### 6.2. Energetic Validation

In this section, we verify the energy consumption during these experiments for the two configurations. An energy-like criterion is proposed:

(43) $$\begin{array}{cc} E & =\sum_{i=1}^{m}\int_{t=0}^{T}\left|PWM^{i}\left(t\right)-1500\right|dt \end{array}$$

where *m* is the number of thrusters, *T* is the time of the experiment, and $PWM^{i}\left(t\right)$ is the PWM inputs of the *i*th thruster.

Table 6 shows the energy consumption of the robot during the three rotation experiments. For u-axis rotation, the attainability of the two configurations was the same, but the energy consumption of $C^{2}$ was lower than that of $C^{1}$. For v-axis and w-axis rotations, the duration of the experiments of $C^{2}$ was longer than that of $C^{1}$, and the energy consumption, therefore, was higher.

Table 7 shows the comparison of the energy consumption of the two configurations with the same time duration. For the v-axis rotation, the energy value of $C^{2}$ was lower than that of $C^{1}$. However, for the w-axis, the energy value of $C^{2}$ was higher. This happened because the robot dived deeper in $C^{2}$ in the experiment of the w-axis rotation, and the robot had to deliver more power to maintain a greater constant depth.

### 6.3. Robustness and Reactive Validation

This section validates the robustness and reactivity of the optimal configuration ($C^{2}$) in comparison to the normal one ($C^{1}$). For robustness, the robot performed a mission, and one or two thrusters were turned off. For the normal configuration $C^{1}$, the mission would fail, and for the optimal configuration $C^{2}$, the mission would be guaranteed. Specifically, for the robustness index, we carried out the following experiments:

1. The cube robot dives to a predefined depth with all motors being in the normal operating conditions;
2. The cube robot dives to the same predefined depth with one vertical motor being stopped;
3. The cube robot dives to the same predefined depth with two vertical motors being stopped;
4. The cube robot dives to the same predefined depth with three motors being stopped (two vertical motors and one arbitrary motor);
5. The cube robot simultaneously dives to the same predefined depth and rotates about the w-axis with three motors being stopped (two vertical motors and one horizontal motor)

For the reactive index, we measured how fast the robot changed missions. The following experiments were carried out:

1. The cube robot goes down to the predefined depth and goes up to another predefined depth, then goes down again to the former predefined depth;
2. In the sequel, the cube robot goes down to the predefined depth, rotates about the u-axis, and after that, rotates about the v-axis. The rotation time of each axis should be 60 s or longer;
3. Next, the cube robot goes down to the predefined depth, rotates about the u-axis, and after that, rotates about the diagonal-axis (diagonal of the cube robot). The rotation time of each axis should be 60 s or longer.

**Figure 27:**
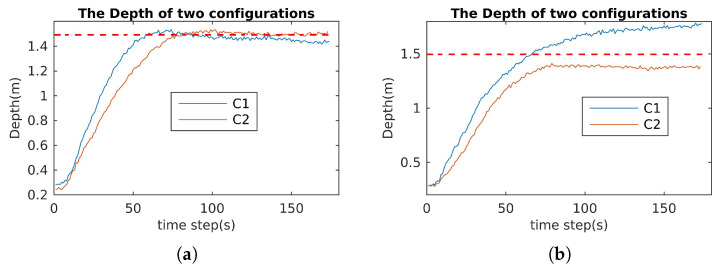
Depth control for $C^{1}$ and $C^{2}$ with one and two motors stopped. (**a**) Depth control of two configurations with one motor stopped. (**b**) Depth control of two configurations with two motors stopped.

**Figure 28:**
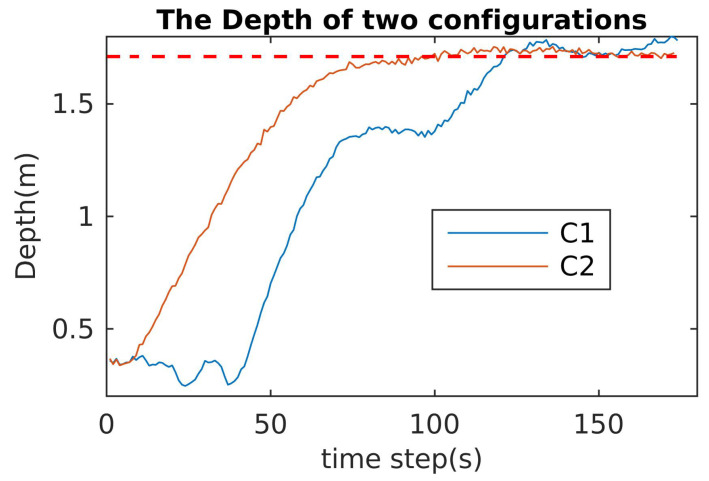
Depth control for $C^{1}$ and $C^{2}$ with three motors stopped.

**Figure 29:**
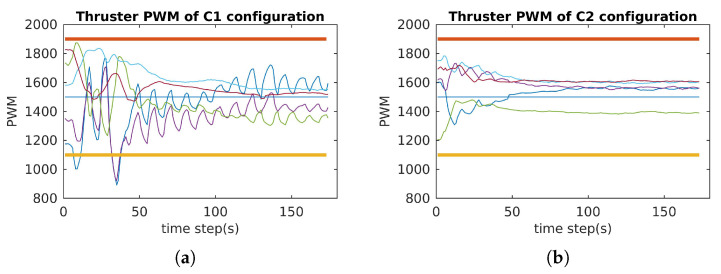
PWM evaluation for $C^{1}$ and $C^{2}$ with 3 motors stopped. (**a**) PWM of $C^{1}$. (**b**) PWM of $C^{2}$.

The experimental results for the robustness validation of $C^{1}$ and $C^{2}$ are shown in Figure 27, Figure 28 and Figure 29. In the case of one or two motors stopped, the depth control performances of $C^{1}$ and $C^{2}$ were almost the same (see Figure 27). The differences are clear in the case of three thrusters stopped (Figure 29): the performance of $C^{1}$ was not guaranteed (Figure 28) and violations of the PWM values occurred (see Figure 29a).

**Figure 30:**
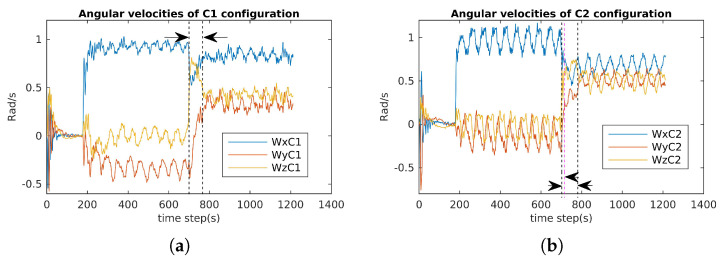
Angular velocity evaluation for $C^{1}$ and $C^{2}$: diving, rotating about the u-axis, and rotating about the diagonal-axis (Wx=p; Wy=q; Wz=r). (**a**) Angular velocities of $C^{1}$. (**b**) Angular velocities of $C^{2}$.

**Figure 31:**
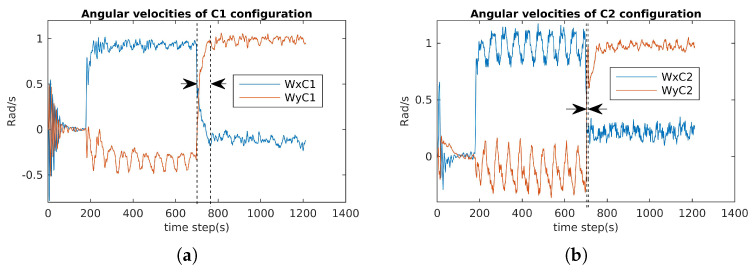
Angular velocity evaluation for $C^{1}$ and $C^{2}$: diving, rotating about the u-axis, and rotating about the v-axis (Wx=p; Wy=q). (**a**) Angular velocities of $C^{1}$. (**b**) Angular velocities of $C^{2}$.

**Figure 32:**
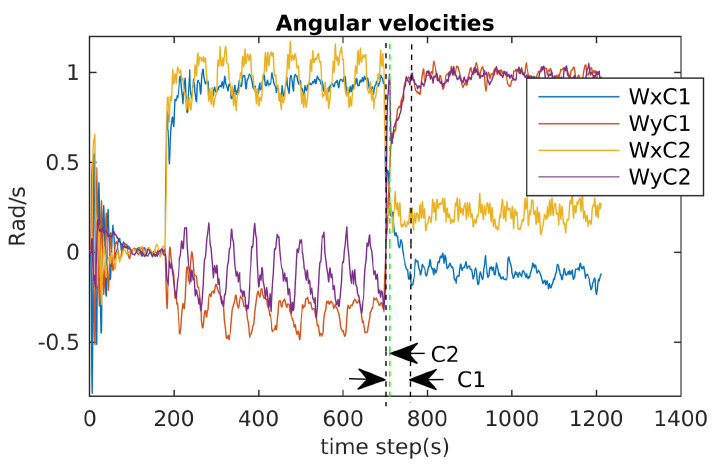
Angular velocity evaluation for $C^{1}$ and $C^{2}$: diving, rotating about the u-axis, and rotating about the v-axis (Wx=p; Wy=q).

The results for the reactive validation are shown in Figure 30, Figure 31 and Figure 32. We measured the reactive time of the angular velocities when the directions of the cube’s actions changed. It is clear that the reactive time of $C^{2}$ was faster than that of $C^{1}$. Specifically, the reactive time is the region formed by the vertical dashed lines in Figure 30, Figure 31 and Figure 32. It is obvious that the reactive time of $C^{2}$ was smaller than that of $C^{2}$ (see Figure 31 and Figure 32).

## 7. Conclusions and Future Work

In this paper, an approach for designing an optimal configuration matrix (which depends on the positions and directions of the thrusters) of overactuated underwater robots was presented. The performance indices (related to manipulability, energy, workspace, reactivity, and robustness) were proposed and analyzed. Specifically, the manipulability index shows the isotropic properties of a robot; the energetic index minimizes the energy consumption under some assumptions; the workspace index is related to the attainable spaces (i.e., the force and torque spaces) of the robot; the reactive index presents how fast the robot changes the direction of the resulting actuation force; finally, the robustness index is related to the capacity of the robot to maintain its performance in the case of failures (i.e., some thrusters are completely stopped). It was formulated as a multi-objective optimization problem. Because the different indices exhibit different magnitudes and physical meanings, the goal-attainment method was chosen to find one Pareto-optimal solution. Simulation and experimental results showed that the performances of the optimal configuration were better than a “normal” configuration, which is often used (thrusters are installed vertically or horizontally). Because of the nonconvexity of the problem, finding all Pareto-optimal solutions, the Pareto front, remains a challenging problem and will be future work. Moreover, a design problem relaxing the assumptions (i.e., perfectly known characteristics of the actuators, pseudo-inverse dispatcher) is also an interesting direction for future research.

## Figures and Tables

**Table 1:** Notations.

**A**	Configuration matrix
$A^{+}$	Moore–Penrose pseudo-inverse of A matrix
$u_{i}$	(3×1) unit vector of the direction of the *i*th thruster
$r_{i}$	(3×1) unit vector of the position of the *i*th thruster
$F_{m}$	(m×1) force vector of m thrusters
$F_{m}^{d}$	(m×1) desired force vector of m thrusters
$F_{m,i}$	Force magnitude of the *i*th thruster
$F_{B}^{d}$	(6×1) desired force (force and torque elements) w.r.t. the body-frame
FB=FΓ	(6×1) resulting force (force and torque elements) w.r.t. the body-frame
$c_{m}$	(m×1) input vector of thrusters
⊗	Cross product
∥·∥	Euclidean norm
${∥\cdot ∥}_{p}$	p-norm
*m*	Number of thrusters
*n*	Number of Degrees of Freedom (DoFs)
F	(3×1) vector of force elements in the resulting force $F_{B}$
Γ	(3×1) vector of torque elements in the resulting force $F_{B}$
D	Dispatcher
$d_{i}$	Distance of the *i*th thruster to the center of the body-frame
cond(A)	Condition number of the matrix A
Vol(.)	Volume of a space
rank(.)	Rank of a matrix

**Table 2:** Desired values of the indices.

Index	Optimal Formula and Condition	Desired Value
$I_{m}^{d}$	$\sigma_{max}=\sigma_{min}$	1
$I_{e}^{d}$	2 $∥A^{+}∥$	1.2248
$\frac{1}{I_{w}^{d}}$	see Equations (35) and (36) and $\frac{1}{I_{w}^{d}}=\frac{1}{I_{wF}^{d}}+\frac{1}{I_{wT}^{d}}$	0.0033
$I_{re}^{d}$	$\frac{1}{\sigma^{max}}$	0.6124

**Table 3:** Configuration matrix in the general case.

Configuration Matrix	Optimal Value	Attainment Factor
$A=\left(\begin{array}{rrrrrrrr} -0.8891 & -0.3645 & 0.5438 & 0.9879 & 0.3134 & 0.0148 & 0.0495 & 0.6090\\ -0.0985 & -0.3036 & -0.5911 & -0.0608 & -0.9493 & 0.0515 & 0.8919 & 0.7158\\ 0.4471 & 0.8803 & 0.5957 & 0.1429 & 0.0260 & 0.9986 & 0.4495 & 0.3417\\ -0.4308 & 0.4701 & -0.8386 & 0.0379 & -0.1336 & 0.5628 & -0.9972 & 0.4758\\ 0.5107 & 0.7561 & -0.4103 & 0.9868 & -0.0712 & -0.8259 & 0.0690 & 0.0149\\ -0.7441 & 0.4554 & 0.3583 & 0.1577 & -0.9885 & 0.0342 & -0.0272 & -0.8794 \end{array}\right)$	$Fval=\left(\begin{array}{c} 1.0000\\ 1.2200\\ 0.0050\\ 0.6124 \end{array}\right)$	0.3896

**Table 4:** Configuration matrix in the given position case.

Configuration Matrix	Optimal Value	Attainment Factor
$A=\left(\begin{array}{rrrrrrrr} 0.0836 & 0.6616 & -0.8122 & 0.4785 & -0.6616 & -0.0836 & -0.4785 & -0.8122\\ 0.7452 & 0.7452 & 0.3337 & 0.3337 & 0.7452 & 0.7452 & 0.3337 & -0.3337\\ 0.6616 & -0.0836 & -0.4785 & -0.8122 & 0.0836 & -0.6616 & 0.8122 & -0.4785\\ -0.8122 & 0.4785 & -0.0836 & -0.6616 & -0.4785 & 0.8122 & 0.6616 & -0.0836\\ -0.3337 & -0.3337 & 0.7452 & 0.7452 & -0.3337 & -0.3337 & 0.7452 & -0.7452\\ 0.4785 & 0.8122 & 0.6616 & -0.0836 & -0.8122 & -0.4785 & 0.0836 & 0.6616 \end{array}\right)$	$Fval=\left(\begin{array}{c} 1.0000\\ 1.2200\\ 0.0050\\ 0.6124 \end{array}\right)$	0.3868

**Table 5:** Comparison between the two configurations ($I_{ro}$ shows the maximum number of thrusters that can fail to make sure that rank(A=6)).

No.	Indices	$C^{1}$	$C^{2}$
1	$I_{m}$	7.12	2.559
2	$I_{e}$	3.32	2.09
3	$I_{w}$	6,511,536.45	10,919,428.13
4	$I_{re}$	4.05	1.56
5	$I_{ro}$	0	2

**Table 6:** Energy consumption of the two configurations.

No.	Rotation	$E_{C^{1}}$	$E_{C^{2}}$
1	u	7.2303 $\times \;10^{4}$	6.9603 $\times \;10^{4}$
2	v	7.5480 $\times \;10^{4}$	1.0590 $\times \;10^{5}$
3	w	3.1637$\;\times \;10^{4}$	7.4350 $\times \;10^{4}$

**Table 7:** Energy consumption of the two configurations with the same time duration.

No.	Rotation	$E_{C^{1}}$	$E_{C^{2}}$
1	v	7.5480 $\times \;10^{4}$	7.2715 $\times \;10^{4}$
2	w	3.1637 $\times \;10^{4}$	3.3312 $\times \;10^{4}$

## Data Availability

Not applicable.

## Abbreviations

The following abbreviations are used in this manuscript:

AS	Actuation System
NGC	Navigation–Guidance–Control
DoFs	Degrees of Freedom
AUVs	Autonomous Underwater Vehicles
CM	Center of Mass
PWM	Pulse-Width Modulation

## Appendix A

**Theorem** **A1.**
*The image of the unit hypersphere under any n×m matrix is a hyperellipsoid.*

**Proof.** Let A be an n×m matrix with rank *r*. Let $A=USV^{T}$ be a singular-value decomposition of A. The left and right singular vectors of A are denoted as $u_{1},u_{2},\ldots ,u_{n}$ and $v_{1},v_{2},\ldots ,v_{m}$, respectively. Since rank(A)=r, the singular values of A have the properties: $\sigma_{1}\geq \sigma_{2}\geq ,\ldots ,\geq \sigma_{r}>0$ and $\sigma_{r+1}=\sigma_{r+2}=,\ldots ,=\sigma_{m}=0$. Let $x=\left(\begin{array}{c} x_{1}\\ \vdots \\ x_{m} \end{array}\right)$ be an unit vector in $R^{m}$. Because V is an orthogonal matrix and $V^{T}$ is also, we have that $V^{T}x$ is an unit vector (it is easy to see that $∥V^{T}x∥=∥x∥$). Therefore, ${\left(v_{1}^{T}x\right)}^{2}+{\left(v_{2}^{T}x\right)}^{2}+,\ldots ,+{\left(v_{m}^{T}x\right)}^{2}=1$. On the other hand, we have $A=\sigma_{1}u_{1}v_{1}^{T}+\sigma_{2}u_{2}v_{2}^{T}+,\ldots ,+\sigma_{r}u_{r}v_{r}^{T}$. Therefore:

(A1) $$\begin{array}{cc} Ax & =\sigma_{1}u_{1}v_{1}^{T}x+\sigma_{2}u_{2}v_{2}^{T}x+,\ldots ,+\sigma_{r}u_{r}v_{r}^{T}x\\ \; & =\left(\sigma_{1}v_{1}^{T}x\right)u_{1}+\left(\sigma_{2}v_{2}^{T}x\right)u_{2}+,\ldots ,+\left(\sigma_{r}v_{r}^{T}x\right)u_{r}\\ \; & =y_{1}u_{1}+y_{2}u_{2}+,\ldots ,+y_{r}u_{r}\\ \; & =Uy \end{array}$$

where $y_{i}$ denotes the $\sigma_{i}v_{i}^{T}x$ and $y=\left(\begin{array}{c} y_{1}\\ \vdots \\ y_{r} \end{array}\right)$. From (A1), we have: ∥Ax∥=∥Uy∥=∥y∥ (since U is an orthogonal matrix). Moreover, y has the following property:

(A2) $$\begin{array}{cc} \; & {\left(\frac{y_{1}}{\sigma_{1}}\right)}^{2}+{\left(\frac{y_{2}}{\sigma_{2}}\right)}^{2}+,\ldots ,+{\left(\frac{y_{r}}{\sigma_{r}}\right)}^{2}=\\ \; & ={\left(v_{1}^{T}x\right)}^{2}+{\left(v_{2}^{T}x\right)}^{2}+,\ldots ,+{\left(v_{r}^{T}x\right)}^{2}\leq 1 \end{array}$$

Specifically:

1. If r=m (of course, we must have m≤n), the equality in Equation (A2) holds, and the image of the unit hypersphere forms the surface of a hyperellipsoid;
2. If r<m, the image of the unit hypersphere corresponds to a solid hyperellipsoid.

This completes the proof.   □
